# Fishbone ingestion is a non-negligible cause of intestinal perforation

**DOI:** 10.3389/fmed.2025.1607262

**Published:** 2025-08-13

**Authors:** Wenwei Zuo, Jiexin Bai, Jing Wei, Chuan Long

**Affiliations:** ^1^Hospital of Chengdu University of Traditional Chinese Medicine, Chengdu, Sichuan, China; ^2^Eye College of Chengdu University of Traditional Chinese Medicine, Chengdu, Sichuan, China

**Keywords:** foreign bodies, intestinal perforation, diagnosis, abdomen, acute

## Abstract

Foreign body ingestion (FBI) is considered a widespread global health concern, with fishbone ingestion (FI) occurring frequently. However, fishbone-induced intestinal perforation (FIIP) remains rare and is frequently overlooked in the initial differential diagnosis. A case involving a 39-year-old patient presenting with acute abdominal pain was diagnosed as FIIP. Initial laparoscopic surgery was followed by a laparotomy for fishbone removal, resulting in a favorable patient recovery. The existing literature on FIIP is reviewed in this article. Reported cases underscore the necessity of prompt identification of the perforation’s cause and the critical role of thorough medical history-taking. Computed tomography (CT) and ultrasonography are considered essential diagnostic tools in confirming the condition. While ultrasonography serves as a rapid, non-invasive preliminary examination, CT is regarded as more accurate and comprehensive. In regions with high fish consumption, FIIP should be considered in adult patients, especially the elderly. Retained fishbones may result in serious complications and should be removed whenever feasible. Clinical education is considered vital in minimizing delays in diagnosis and treatment. The least invasive treatment strategy should be selected according to the patient’s clinical status.

## 1 Introduction

Currently, over 4,000 peer-reviewed publications worldwide have reported clinical case reports and case series related to foreign body ingestion (FBI). Most FBIs are expelled spontaneously or result in minor mucosal injuries. However, in rare cases, sharp foreign bodies may lead to gastrointestinal perforation, severe gastrointestinal hemorrhage, sepsis, hepatic abscess, or even death (1–3). It is estimated that approximately 1% of all ingested foreign bodies result in complications, such as mucosal injuries, impaction, or perforation (4). Among these complications, up to 63% are attributed to fish bones, highlighting the clinical relevance of fishbone-related injuries. While intestinal perforation is one of the rarest outcomes, fishbone-induced intestinal perforation (FIIP) remains a distinct and potentially life-threatening condition that warrants heightened clinical awareness (5). Gastrointestinal perforations caused by the FBI have not received sufficient attention. In numerous low- and middle-income countries globally, fish has emerged as a principal source of animal protein and essential nutrients (6, 7). A case of terminal ileal perforation resulting from accidental fishbone ingestion (FI) is presented. Given the absence of prior comprehensive reviews on FIIP, a summary of all relevant studies is warranted. A comprehensive literature review was conducted, focusing on clinical characteristics, patient demographics, diagnostic imaging, surgical and conservative management strategies, and geographical patterns, to enhance diagnostic accuracy and therapeutic outcomes.

## 2 Case presentation

**Figure 1:**
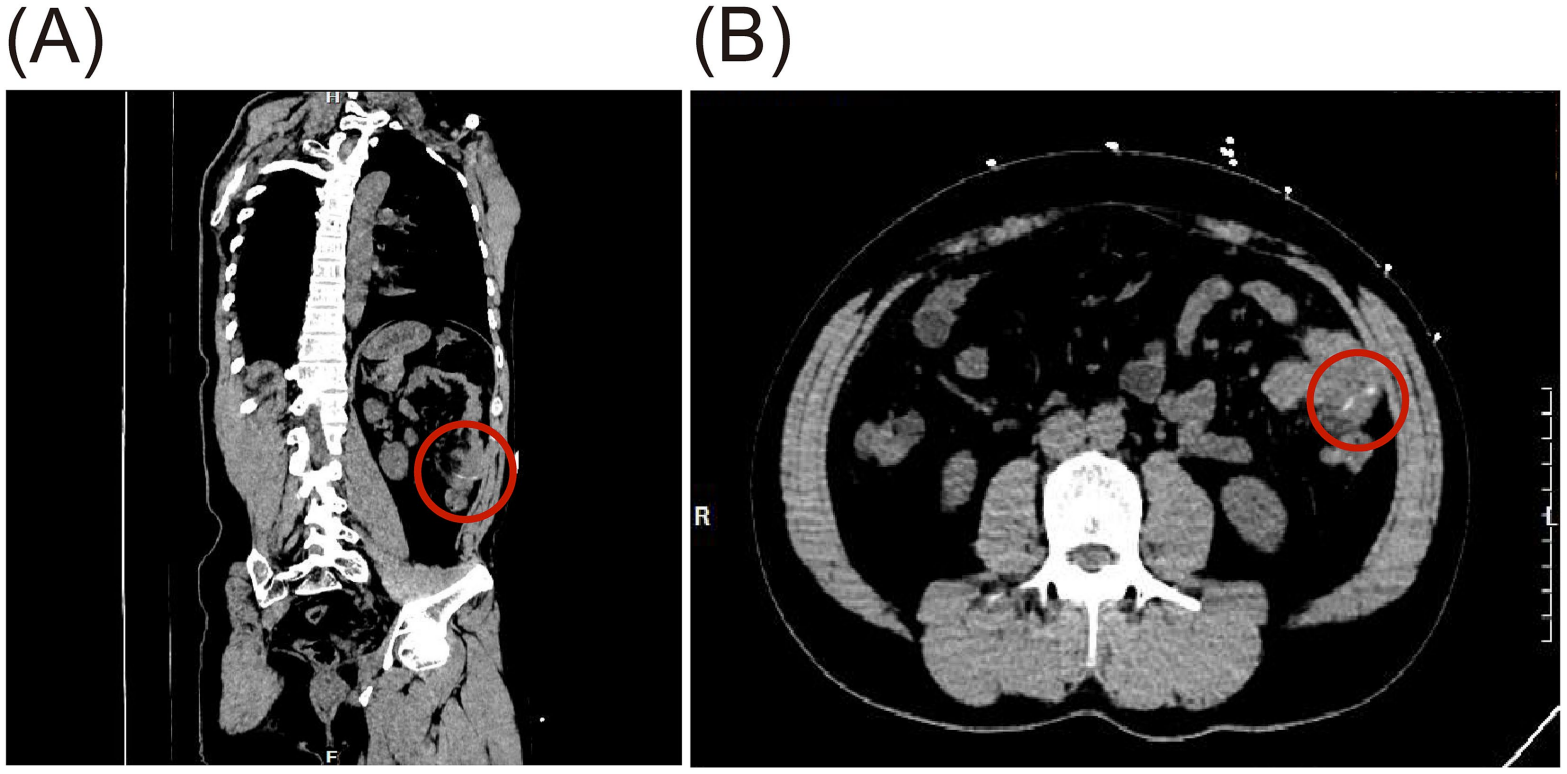
Preoperative CT imaging of small bowel perforation caused by FI, showing a linear hyperdense foreign body about 3 cm long in the small intestine (red circle). **(A)** Oblique sagittal plane. **(B)** Coronal plane.

**Figure 2:**
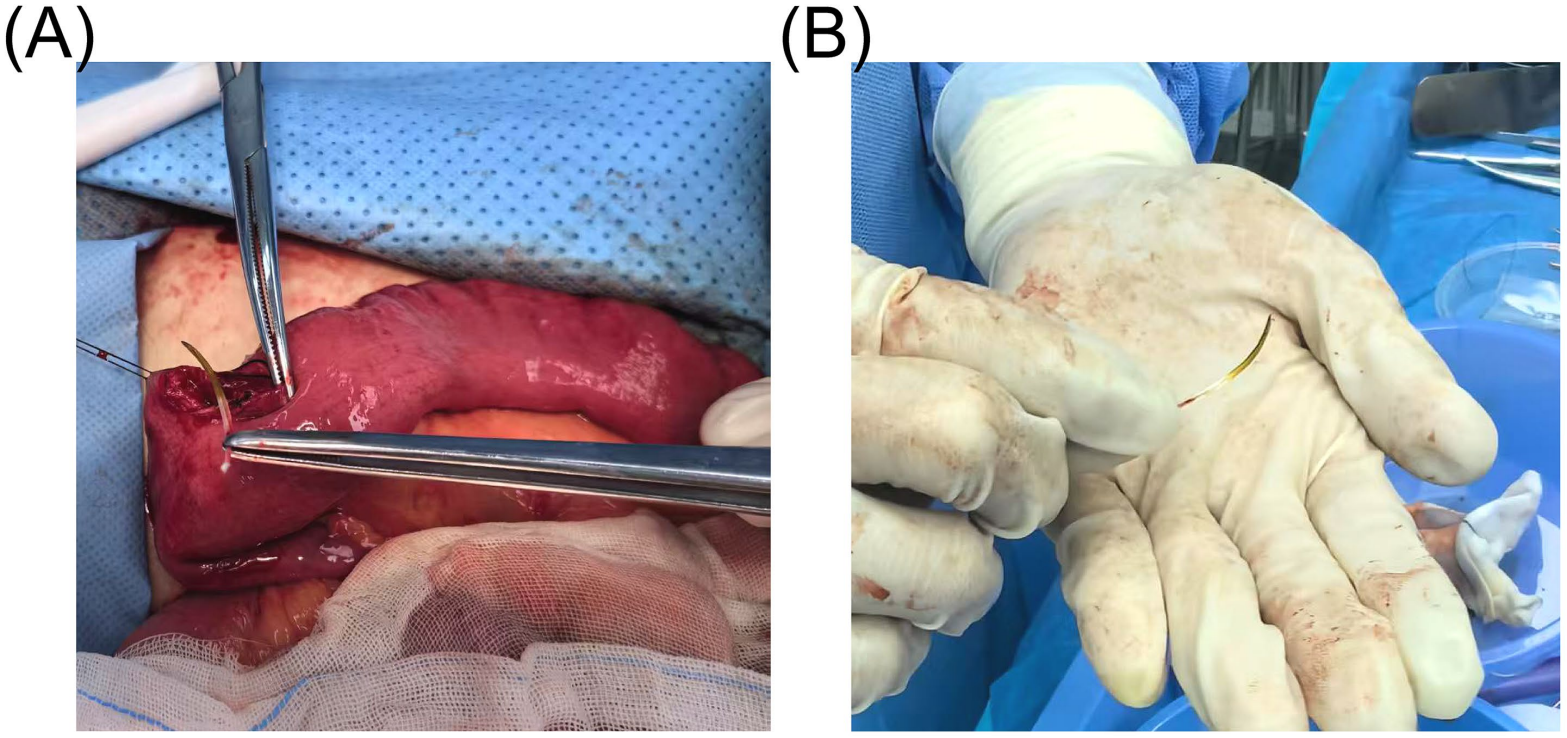
The fishbone that had perforated the small intestine was removed during surgery. **(A)** Intraoperative findings revealed that the fishbone had perforated the small intestine, and the wound was closed using 4-0 absorbable sutured. **(B)** The fishbone was successfully removed and displayed.

A 39-year-old male patient was admitted with a 3-day history of abdominal distension. He had no significant medical history and no prior abdominal surgeries. During this time, he reported decreased appetite, absence of bowel movements for 2 days, fever peaking at 38.5°C, and chills. He attributed all symptoms to an upper respiratory tract infection. Upon admission, nausea, vomiting, and diarrhea were denied. Physical examination revealed abdominal distension, fever (38.6°C), diffuse tenderness in the mid-to-upper abdomen, tympany on percussion, abdominal guarding, and absence of rebound tenderness. Laboratory tests demonstrated leukocytosis (WBC count: 11.78 × 10^9^/L), neutrophilia (NEUT count: 8.06 × 10^9^/L), and elevated C-reactive protein (CRP) levels (244.75 mg/L). Computed tomography (CT) scan revealed scattered gas and fluid in parts of the mid-to-upper small intestine without notable dilation, areas of increased and hazy fat density with patchy shadows in the abdomen, adjacent peritoneal thickening, and an irregular arc-shaped hyperdense structure partially penetrating the intestinal wall was identified within a segment of the left mid-abdominal small intestine (Figure 1), measuring approximately 3 cm in length (Figure 1). The patient subsequently recalled ingesting a fishbone of similar length 3 days prior. Emergency laparoscopic exploration was initiated and subsequently converted to open laparotomy. Intraoperatively, marked dilatation of the small intestine was observed, without bile leakage or purulent fluid in the peritoneal cavity. An embedded fishbone was found lodged in the terminal ileum (2 cm from the ileocecal valve), with its tip (approximately 0.5 cm long) protruding through the intestinal wall, while the rest remained in the intestinal lumen. Mild adhesions, localized inflammatory reaction, and minimal purulent exudate were noted at the site of perforation, with no evidence of abscess formation nearby. Through limited enlargement of the perforation site, a 3-cm-long fishbone was completely extracted (Figure 2). The perforation site was closed with four 4-0 absorbable sutures (Figure 2). A peritoneal drain was placed adjacent to the perforation site in the peritoneal cavity for drainage. An additional drain was positioned in the pelvic cavity to prevent postoperative inflammatory exudate accumulation, given that the pelvis is the lowest point of the peritoneal cavity when upright. No evidence of ischemic necrosis in the small intestine was observed, hence bowel resection was unnecessary. The procedure was uneventful. The fishbone was successfully removed, and the intestinal defect was repaired. The patient passed flatus on postoperative day 2, resumed oral intake thereafter, had a bowel movement on postoperative day 5, and was discharged on day 7.

## 3 Discussion

FBI may result in clinical outcomes ranging from mild symptoms to life-threatening complications (8), most frequently observed in children between 6 months and 6 years of age (9). Among adults, aside from food bolus impaction secondary to underlying gastrointestinal pathology, FBI is more frequently reported in elderly individuals, patients with intellectual disabilities, individuals with alcohol use disorder, incarcerated populations, and those with psychiatric conditions (10). The majority of FBI cases are asymptomatic, with gastrointestinal perforation constituting a rare complication, occurring in less than 1% of cases (11, 12). Once perforation occurs, the risk of localized infection, gastrointestinal hemorrhage, abscess formation, peritonitis, and sepsis increases significantly. Approximately 1 to 14% of patients may require surgical intervention (11, 13–15). Ingestion of sharp objects—such as animal bones, sewing needles, and toothpicks—represents the major risk factor for gastrointestinal perforation caused by FBI (12).

The reported frequency of FBI varies considerably across published studies. In adults, fishbones constitute the most frequently ingested food-related foreign bodies, accounting for 9 to 45% of cases (16). This variability is likely attributable to regional dietary practices, increased consumption of fish as a protein source, and the recognized health benefits associated with fish consumption (17, 18). Most fishbones are sharp, small, and resistant to gastric acid, making them particularly prone to causing intestinal perforation following ingestion. FIIP typically occurs unintentionally and without the patient’s awareness. When symptoms related to gastrointestinal perforation emerge, patients often have difficulty recalling any history of FBI. In the case presented, the patient initially sought treatment at a local clinic for fever, abdominal distension, and anorexia over a 3-day period, during which an upper respiratory tract infection was diagnosed. The accidental ingestion of a fishbone was recalled only after the CT findings were disclosed. Based on both the literature review and the current case, a multidimensional analysis of FIIP was conducted (Table 1).

**Table 1:** Previously published case reports and case series of fishbone-induced intestinal perforation, categorized by country, patient age, diagnostic method, type of fish, location, onset of symptoms or time of ingestion, prognosis, and therapeutic interventions (*N* = 58).

Authors	Country and region	Year of publication	Age	Method of confirmation	Fish species	Perforation site	Secondary segment	Fishbone length (cm)	Time of fishbone ingestion or onset of symptoms	Main intervention	Outcome (recovered/deceased)
Goh et al. (46)	Singapore	2005	32	ST (Lap)	Unknown	SI	Duo	3.0	5 days	ST (Lap)	Recovered
Völkl et al. (51)	Germany	1997	57	CT	Unknown	SI	Duo	2.0	10 days	Duodenoscope	Recovered
Chen et al. (89)	China, Taiwan	2011	59	ST (Lap)	Unknown	SI	Duo	4.0	2 weeks	ST (Lap)	Recovered
Brandão et al. (20)	Portugal	2010	61	CT	Unknown	SI	Duo	Unknown	3 days	Con Tx (Abs + anticoagulation)	Recovered
Wang et al. (21)	United States	2020	63	CT	Unknown	SI	Duo	Unknown	1 month	ST (Lap)	Recovered
Wang et al. (41)	China	2021	68	CT	Unknown	SI	Duo	3.0	14 days	ST (LS to Lap)	Recovered
Yasuda et al. (140)	Japan	2010	73	CT	Marbled sole	SI	Duo	4.0	3 days	ST (Lap)	Recovered
Nishino et al. (104)	Japan	2012	77	CT	Unknown	SI	Duo	3.0	3 days	Duodenoscope	Recovered
Shibuya et al. (52)	Japan	2008	33	Double-balloon endoscopy	Eel	SI	Jej	1.1	8 months	Double-balloon endoscopy	Recovered
Lin and Wu (48)	China, Taiwan	2012	45	CT	Unknown	SI	Jej	3.0	3 days	ST (Lap)	Recovered
Dural et al. (118)	Turkey	2016	52	ST (LS)	Unknown	SI	Jej	Unknown	3 days	ST (LS)	Recovered
Jallali et al. (143)	United States	2024	55	CT	Unknown	SI	Jej	2.3	2 days	ST (LS)	Recovered
Choi (14)	South Korea	2014	57	ST (Lap)	Japanese red rockfish	SI	Jej	2.7	3 days	ST (Lap)	Recovered
Rodríguez-Hermosa et al. (124)	Spain	2009	58	ST (Lap)	Unknown	SI	Jej	2.0	Unknown	ST	Deceased
Alkhatib et al. (53)	United States	2013	67	Double-balloon endoscopy	Cyprinus carpio	SI	Jej	2.2	7 days	Double-balloon endoscopy	Recovered
Mora-Guzmán et al. (40)	Spain	2019	74	CT	Unknown	SI	Jej	Unknown	4 weeks	Con Tx (Abs)	Recovered
Drakonaki et al. (50)	Greece	2010	78	Ultrasonography + surgical exploration	Unknown	SI	Jej	3.5	3 days	ST (Lap)	Recovered
Dugas et al. (144)	France	2005	81	CT	Unknown	SI	Jej	Unknown	2 days	ST (Lap)	Recovered
Chiu et al. (22)	China, Taiwan	2014	83	CT	Unknown	SI	Jej	2.5	1 day	ST (Lap)	Recovered
Guillén-Paredes et al. (145)	Spain	2010	35	CT	Dicentrarchus labrax	SI	Ile	4.0	1 week	ST	Recovered
Kuo (23)	China, Taiwan	2012	44	CT	Unknown	SI	Ile	2.6	1 day	Con Tx (Abs)	Recovered
Chandrasinghe et al. (146)	Sri Lanka	2015	45	ST (LS)	Unknown	SI	Ile	2.0	3 days	ST (LS)	Recovered
Saunders et al. (28)	England	2014	46	ST (LS)	Unknown	SI	Ile	Unknown	3 days	ST (LS to Lap)	Recovered
Wu (147)	China	2014	48	CT	Unknown	SI	Ile	3.5	5 days	ST (Lap)	Recovered
Song et al. (47)	China	2020	57	ST (Lap)	Argyrosomus argentatus	SI	Ile	1.7	3 days	ST (LS to Lap)	Recovered
Hsu et al. (29)	China, Taiwan	2005	62	ST (Lap)	Gray snapper	SI	Ile	2.5	5 days	ST (Lap)	Recovered
Zhao et al. (148)	China	2019	68	CT	Unknown	SI	Ile	Unknown	1 month	ST (Lap)	Recovered
Mutlu et al. (149)	Turkey	2012	69	CT	Unknown	SI	Ile	Unknown	2 days	ST	Recovered
Hassani et al. (150)	Morocco	2013	70	CT	Unknown	SI	Ile	Unknown	2 days	ST (Lap)	Recovered
Fantola et al. (151)	France	2011	75	ST (LS)	Unknown	SI	Ile	Unknown	Unknown	ST (LS)	Recovered
Bhatia et al. (24)	England	2006	85	ST (Lap)	Rainbow trout	SI	Ile	4.0	3 days	ST (Lap)	Recovered
Masuya et al. (58)	Japan	2019	13	Ultrasonography + CT	Sea bream	SI	Ile, MD	2.0	4 days	ST (LS)	Recovered
Wong et al. (30)	Brunei	2005	21	ST (Lap)	Unknown	SI	Ile, MD	Unknown	1 day	ST	Recovered
Daniele et al. (152)	Australia	2015	42	ST (LS)	Unknown	SI	Ile, MD	Unknown	1 day	ST (LS)	Recovered
Wong et al. (30)	Brunei	2005	49	ST	Unknown	SI	Ile, MD	Unknown	3 days	ST	Recovered
Mouawad et al. (31)	United States	2013	52	ST (LS)	Unknown	SI	Ile, MD	Unknown	Unknown	ST (LS)	Recovered
Natsuki et al. (42)	Japan	2020	54	CT	Unknown	SI	Ile, MD	2.0	1 month	ST (LS)	Recovered
Gonçalves et al. (60)	Portugal	2016	61	CT	Unknown	SI	Ile, MD	2.5	1 day	ST (LS)	Recovered
McDowell and Bush (32)	United States	1982	72	ST	Unknown	SI	Ile, MD	3.0	2 days	ST	Recovered
Ward et al. (94)	United States	2012	28	CT	Northern pike	SI	Unknown	2.0	1 day	ST (Lap)	Recovered
Wu and Chiu (139)	China, Taiwan	2020	34	CT	Unknown	SI	Unknown	2.8	1 day	ST (LS)	Recovered
Taguchi and Kitagawa (112)	Japan	2019	73	CT	Yellowtail fish	SI	Unknown	5.0	2 month	Con Tx	Recovered
Lunsford et al. (49)	Tunisia	2011	76	ST (LS)	Unknown	SI	Unknown	3.0	1 day	ST (LS)	Recovered
Lim and Siew (95)	Singapore	2011	81	CT	Unknown	SI	Unknown	5.0	2 month	Con Tx	Recovered
Zhou et al. (153)	China	2023	41	CT	Unknown	LI	App.	3.0	Unknown	ST (LS)	Recovered
Kuwahara et al. (19)	Japan	2019	55	CT	Unknown	LI	Cec	2.0	Unknown	ST (LS)	Recovered
Ishimura et al. (154)	Japan	2006	79	CT	Sea bream	LI	Cec	3.0	2 days	ST (Lap)	Recovered
Yamamoto et al. (155)	Japan	2015	69	CT	Sebastes (fried)	LI	CO (AC)	2.0	8 weeks	ST (Lap)	Recovered
Chiu et al. (156)	China, Taiwan	2009	60	CT	Unknown	LI	CO (TC)	3.5	4 weeks	ST (Lap)	Recovered
Yuan et al. (141)	China	2024	53	Unknown	CT	LI	CO (TC)	3.0	1 month	ST (LS)	Recovered
Ueda et al. (82)	Japan	2024	87	CT	Unknown	LI	CO (SC)	3.0	1 day	Colonoscopy	Recovered
Saleem et al. (157)	Kuwait	2023	32	ST (Lap)	Unknown	LI	CO (SC)	Unknown	10 days	ST (LS to Lap)	Recovered
Cho (158)	S. Korea	2014	42	CT	Unknown	LI	CO (SC)	3.5	1 week	ST (intraoperative cystoscopic removal)	Recovered
Watanabe et al. (108)	Japan	2010	61	CT	Unknown	LI	CO (SC)	3.0	6 days	Endoscopic extraction	Recovered
Endo et al. (159)	Japan	2018	62	CT	Unknown	LI	CO (SC)	Unknown	Unknown	ST	Recovered
Hawkyard et al. (160)	England	1931	62	ST (Lap)	Unknown	LI	CO (SC)	5.1	3 days	ST (Lap)	Recovered
Fang et al. (109)	China	2017	68	CT	Unknown	LI	CO (SC)	5.0	1 day	Colonoscopy	Recovered
Yamashita et al. (161)	Japan	2022	64	CT	Unknown	LI	Rectosigmoid	Unknown	Unknown	ST	Recovered

CT, computed tomography; MD, Meckel’s diverticulum; Con Tx, conservative treatment; ST, surgical treatment; LS, laparoscopic surgery; Lap, laparotomy; Abs, antibiotics; SI, small intestine; Duo, duodenum; Jej, jejunum; Ile, ileum; LI, large intestine; Cec, cecum; CO, colon; TC, transverse colon; SC, sigmoid colon; App., appendix; AC, ascending colon.

From 1949 to 2025, a literature search was conducted using search engines including PubMed, Embase, MEDLINE, CINAHL, Ovid, and Cochrane databases. The search terms included “fish bone,” “fishbone,” “fish*,” “seafood,” “intestinal perforation,” “bowel perforation,” “foreign body,” “ingestion,” “peritoneal abscess,” “intestinal injury,” “perforation*,” and “intestin*.” Only articles specifically diagnosing intestinal perforation caused by fishbones were included in the study. Patient characteristics were evaluated. Clinical and imaging findings, subsequent management, and prognosis were determined. Full-text articles of all articles meeting the inclusion criteria were obtained and cross-checked for their references. A summary and analysis of all available data were performed.

### 3.1 Diagnostic methods of FIIP

#### 3.1.1 Medical history, physical examination, and laboratory tests

With regard to medical history, most patients—particularly elderly individuals—are unable to recall the unintentional ingestion of fishbones, significantly complicating the diagnostic process (19). FIIP commonly presents with nonspecific symptoms, including abdominal pain, distension, fever, nausea, anorexia, rebound tenderness, and abdominal rigidity. Laboratory tests typically reveal elevated inflammatory markers in most FIIP patients (20–22), such as increased WBC counts and CRP levels (23, 24). In the absence of a clear ingestion history, acute abdominal symptoms are frequently attributed to alternative conditions such as cholecystitis, appendicitis, peptic ulcer disease, or intra-abdominal malignancies (25–27). In our FIIP case series, four patients were initially misdiagnosed with acute appendicitis (28–31). While others were preoperatively misdiagnosed with colonic diverticulitis (24) or ruptured abdominal aortic aneurysm (32). Despite limitations arising from patient recall bias and the nonspecific clinical presentation, identifying high-risk dietary behaviors and early subtle manifestations through meticulous history-taking remains essential, particularly in regions where fish constitutes a dietary staple. A systematic approach to clinical history, combined with appropriate diagnostic imaging, is crucial for reducing delays in diagnosis.

#### 3.1.2 Imaging examinations of FIIP

##### 3.1.2.1 X-ray

**Figure 3:**
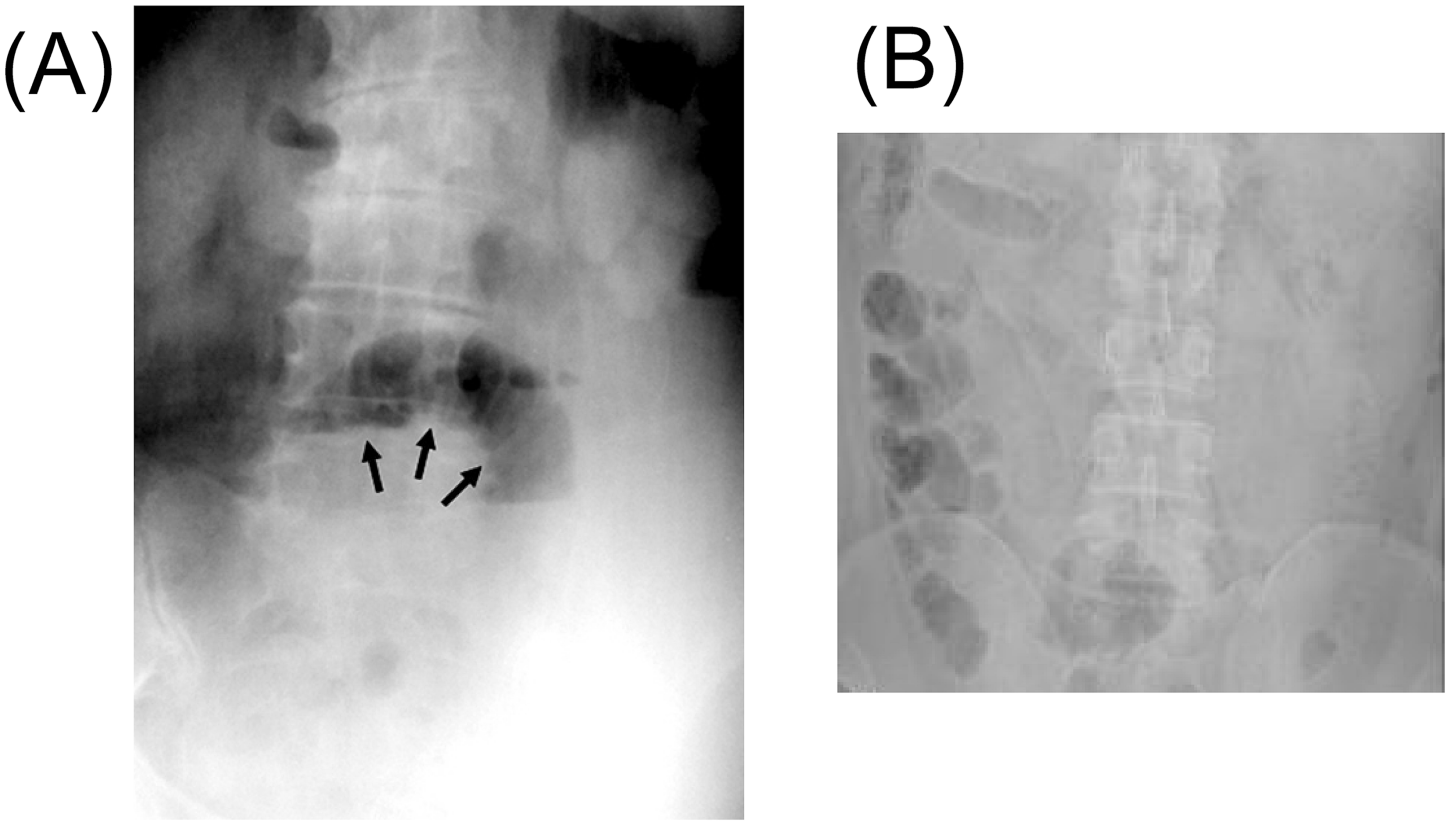
The figure shows the X-ray after intestinal perforation caused by a fishbone. **(A)** The supine abdominal plain film shows dilated small bowel loops, suggesting the possible presence of small bowel obstruction. There is no obvious radiopaque foreign body (black arrow). Adapted from reference (50) with permission from Galenos. **(B)** The abdominal X-ray shows the formation of a localized central intestinal obstruction. Adapted from reference (139) with permission from Springer Nature.

X-ray can be utilized for the detection of ingested foreign bodies and is particularly useful for identifying metallic objects (33–35), bone tissue (36), and pneumoperitoneum (37). Relative to chicken bones, which are almost always radiopaque (38), the radiolucency of fishbones varies by species (39). Fishbones are typically small and slender, and the perforations they cause are often minute. Even when radiopaque, fishbones may become obscured by surrounding fibrin or omental tissue following intestinal perforation. Additionally, perforation sites are often associated with soft tissue swelling, inflammatory changes, abscess formation, and fluid extravasation (29, 40), further masking the subtle osseous signal of the fishbone on radiographs (Figure 3). Consequently, abdominal radiographic findings are frequently unremarkable (41, 42). In a prospective study involving 358 patients with suspected FI, plain radiography demonstrated a sensitivity of only 32% (43). Therefore, the diagnostic utility of plain abdominal X-rays in detecting ingested fishbones is considered limited and unreliable.

##### 3.1.2.2 CT scan

**Figure 4:**
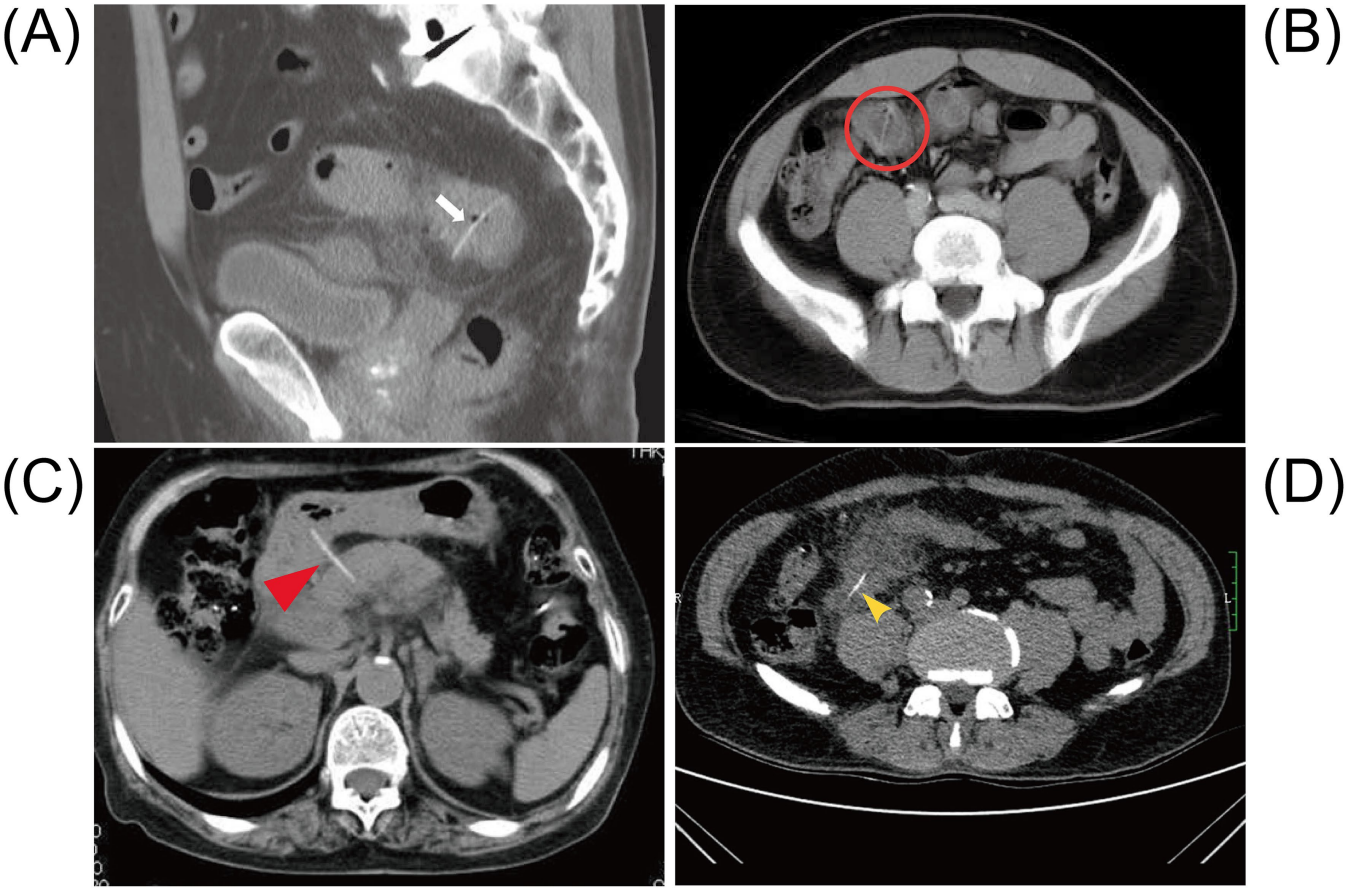
Abdominal CT after intestinal perforation caused by a fishbone. **(A)** The abdominal CT shows a radiopaque linear foreign body penetrating through the ileocecal wall (white arrow). Adapted from reference (108) with permission from J-STAGE. **(B)** The non-contrast abdominal CT shows a 26-mm long radiolucent linear shadow located within thickened bowel walls at both ends in the distal ileum (red circle). Adapted from reference (23) with permission from Baishideng. **(C)** The CT scan reveals a linear calcified body that appears to penetrate the posterior wall of the duodenal bulb and extends into the head of the pancreas. Adapted from reference (140) with permission from Springer Nature. **(D)** The abdominal CT shows patchy exudation and high-density shadow stripes in the right lower abdomen (yellow arrow). Adapted from reference (141) with permission from Springer Nature.

CT examination has played a significant role in the diagnosis of FIIP. Among the 58 cases we collected, over half of the patients were diagnosed with FIIP through CT (*n* = 58, 35/58, 60.3%). Most patients underwent an abdominal CT scan without oral contrast administration. This approach is also supported by previous studies, as the use of contrast agents may delay diagnosis and subsequent intervention (44). Additionally, intravenous contrast may lead to misinterpretation of fishbones as vascular structures, while oral contrast can obscure radiopaque fishbones within the gastrointestinal lumen (45). On CT imaging, perforation caused by foreign body impaction is typically a gradual process; therefore, the presence of free intraperitoneal air is uncommon. Instead, a localized inflammatory response with fibrin deposition frequently occurs at the perforation site. As a result, free air is often confined and manifests as small gas bubbles or pockets in the mesentery near the site of perforation, forming small gas pockets. As observed in the present case, localized accumulation of small bowel gas and fluid was evident. On non-contrast CT scans, fishbones often appear as linear hyperdense structures (46). Whereas on contrast-enhanced CT, they are visualized as more distinct high-density linear shadows (47). Additionally, CT may also show increased density of peritoneal fat, thickening of the peritoneum and bowel wall (48), inflammatory changes, edema (40, 49), abscess formation (14), and even obstruction (50) (Figure 4). CT scans can generally estimate the approximate length and anatomical location of the fishbone. However, definitive identification requires surgical retrieval.

##### 3.1.2.3 Endoscopy

Endoscopy is frequently utilized to evaluate symptoms resulting from FBI, retrieval is generally successful when ingestion is confirmed, particularly in cases where computed tomography fails to identify the fishbone. When the foreign body is retained within the proximal gastrointestinal tract, endoscopic diagnosis and intervention are more feasible. However, endoscopic management of intestinal perforation secondary to FBI has rarely been reported (51–53) (Table 1). This approach is typically reserved for patients who are asymptomatic at the time of presentation.

##### 3.1.2.4 Ultrasound

**Figure 5:**
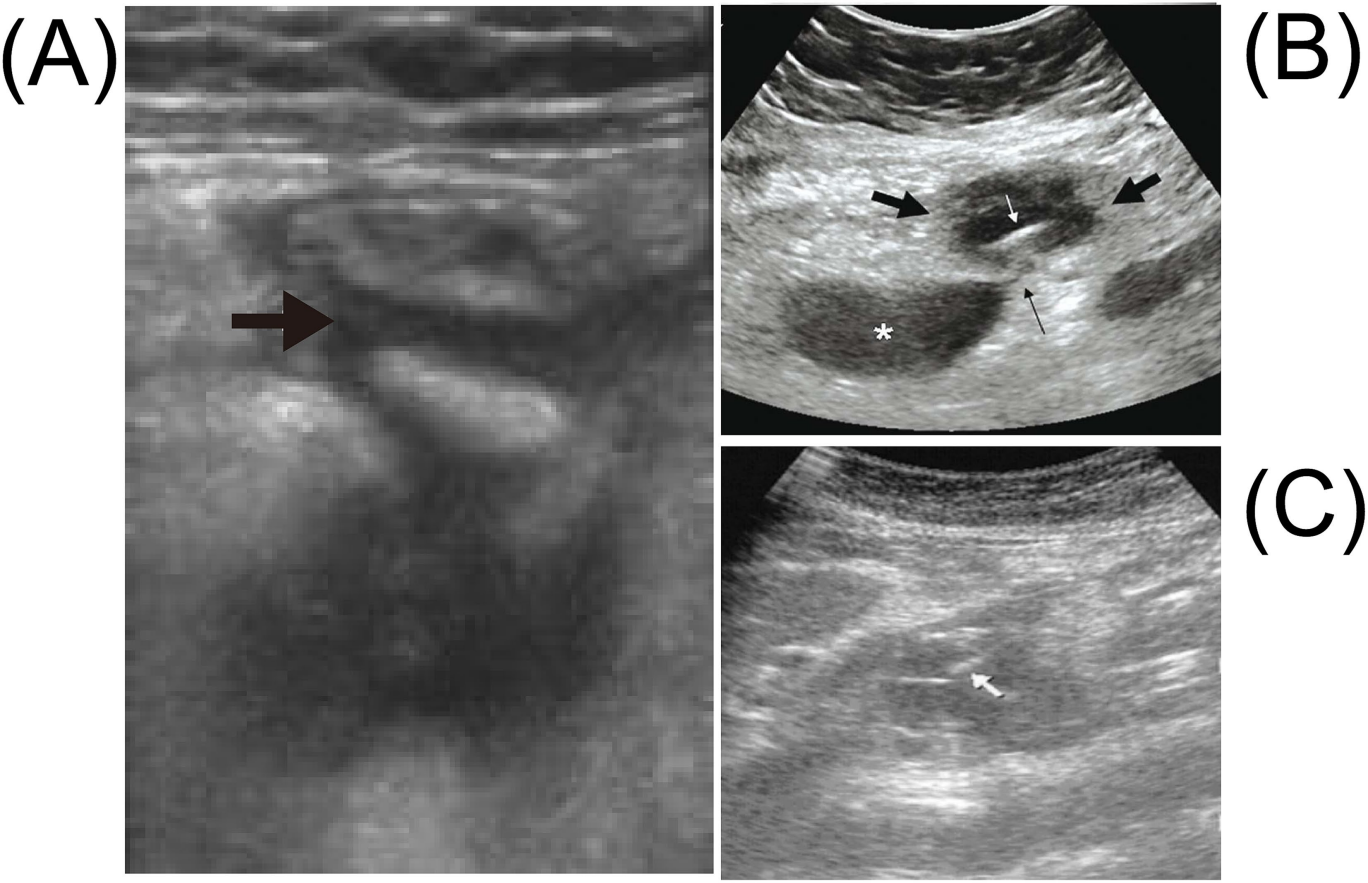
Abdominal ultrasound for intestinal perforation caused by a fishbone shows the following. **(A)** The abdominal ultrasound examination reveals fluid accumulation in the right lower abdomen (black arrow). Adapted from reference (29) with permission from Elsevier. **(B)** The transverse sonographic image of the right periumbilical region shows a hypoechoic mass with a maximum diameter of 4.5 cm (thick black arrow), containing a thin linear echogenic structure measuring 3.7 cm in length (white arrow), representing the fishbone. There is also a hypoechoic sentinel loop of small intestine (asterisk) adjacent to the mass without peristalsis, connected to it via a 2-mm-wide linear hypoechoic sinus tract (thin black arrow). Note the intense echogenicity of the omental fat surrounding the lesion. Adapted from reference (50) with permission from Galenos. **(C)** The abdominal ultrasound shows a hypoechoic mass around the pancreas, with linear echogenic structures around the pancreas (white arrow). Adapted from reference (142) with permission from Oxford Academic.

Foreign bodies, including radiolucent materials such as fishbones and toothpicks, can be identified using ultrasonography due to their high echogenicity and characteristic posterior acoustic shadowing (54). Nevertheless, the widespread adoption of multidetector CT in emergency departments has restricted ultrasound’s role in assessing acute abdominal pain patients. Ultrasonography offers several advantages over CT, including real-time imaging capability, repeatability, portability, cost-effectiveness, absence of ionizing radiation, and the ability to target symptomatic abdominal regions through palpation-guided scanning (55). Ultrasound is generally effective in assessing gastrointestinal perforations, perilesional tissue changes, and luminal contents in superficial intestinal loops or colonic segments. However, evaluation of deeper anatomical structures may be limited (56). Advances in ultrasonographic technology have significantly enhanced diagnostic capabilities, improving image resolution in intestinal evaluations—even in obese patients or when imaging deeper abdominal regions (57) (Figure 5). FBI is particularly common among pediatric populations. As a radiation-free modality, ultrasound holds unique diagnostic value in children, enabling bedside localization of ingested foreign bodies and facilitating prompt surgical removal. Previous studies have demonstrated the diagnostic accuracy of ultrasound in detecting fishbones, typically visualized as linear hyperechoic structures or calcifications, and in identifying associated findings such as intra-abdominal fluid or masses (29, 50, 58, 59). In clinical and emergency settings, the speed, accessibility, radiation-free nature, and diagnostic precision of ultrasound make it an effective first-line imaging modality, particularly in urgent presentations of acute abdominal pain. However, when patient history is unclear, physical findings are nonspecific, and imaging modalities fail to identify the fishbone, laparoscopic or open surgical exploration is required to confirm the diagnosis of FIIP (49, 60).

In conclusion, the timely selection of appropriate diagnostic modalities is essential for the effective management of acute abdominal conditions. In emergency settings, ultrasound serves as an excellent first-line imaging modality, particularly when readily available in the emergency department (61). When performed by experienced operators, ultrasound can effectively identify linear hyperechoic foreign bodies, such as fishbones, and is highly sensitive in detecting abscesses and free intra-abdominal fluid. However, its diagnostic accuracy may be compromised by the presence of intestinal gas, potentially reducing its sensitivity for detecting foreign bodies. Despite these limitations, ultrasonography remains a valuable, non-invasive, radiation-free, and rapid screening tool, especially during the initial evaluation of suspected gastrointestinal perforation.

However, CT remains the most effective imaging modality for diagnosing gastrointestinal foreign bodies. It offers superior spatial resolution and is particularly advantageous for detecting high-density foreign bodies, as well as assessing associated inflammatory changes, abscess formation, and perforation. Its diagnostic sensitivity far exceeds that of X-rays. CT also plays a critical role in determining the necessity of surgical intervention (62). Compared to X-ray, which is typically used for initial evaluation, CT provides more detailed and accurate information regarding the location and impact of foreign bodies.

In certain cases, plain radiography may be performed for basic assessment—particularly to evaluate the presence of free intraperitoneal air or gastrointestinal perforation. However, its sensitivity for detecting fishbones is relatively low, and it is limited in visualizing foreign bodies, especially in the absence of free gas.

When retained fishbones are suspected in the upper gastrointestinal tract (e.g., the esophagus or duodenum), or in the colon and rectum, endoscopy serves as an effective diagnostic and therapeutic modality for localization and retrieval. In situations where imaging and endoscopic findings are inconclusive, or the patient’s clinical condition is unstable and the diagnosis remains uncertain, exploratory laparotomy should be considered as the definitive diagnostic and therapeutic approach.

### 3.2 Age distribution and time to presentation of FIIP

**Figure 6:**
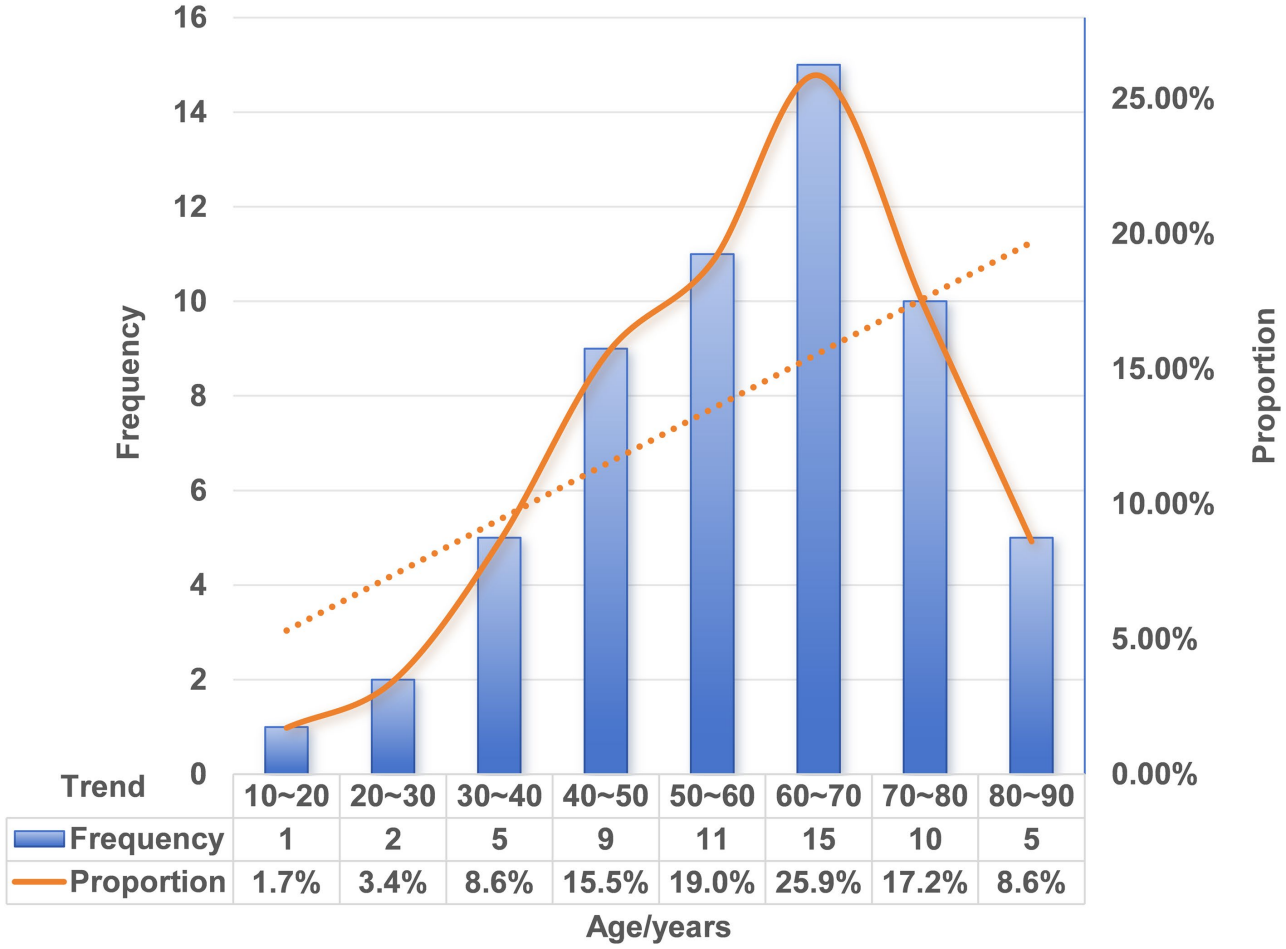
Age frequency histogram with trend line for FIIP.

Extensive epidemiological data have shown that FBI is most prevalent among children aged 5 years or younger, accounting for approximately 75% of documented cases (63). In contrast, the incidence of FIIP is higher among older adults than in pediatric populations (64–66). We conducted a statistical review of patient age in published cases of FIIP. Notably, no cases were reported in infants or toddlers. The youngest affected patient was 13 years old, and the oldest was 87. The age distribution demonstrated right skewness, with a marked predominance in older adults (Figure 6 and Table 1). The absence of FIIP in very young children may be attributed to close adult supervision during fish consumption, including removal of fishbones or provision of boneless fish. Moreover, children possess relatively larger tonsils and smaller oral cavities compared to adults, anatomical features that may predispose them to oropharyngeal foreign body impaction rather than gastrointestinal perforation. Existing evidence suggests that younger children are more likely to seek medical attention promptly, whereas elderly individuals often delay seeking care (67). Our case analysis corroborated previous findings, indicating that adults typically postpone medical evaluation until approximately 3 days after FI or symptom onset (*n* = 51, 35/51, 68.6%). The longest reported interval from ingestion to perforation extended up to 8 months (Table 1). This delay may reflect an underestimation of the clinical risks associated with FI or a failure to recall the ingestion event. Furthermore, elderly patients, who commonly experience tooth loss or use dentures, exhibit reduced tactile sensitivity and increased intestinal fragility with age. Consequently, foreign body ingestion in adults is more likely to remain undetected compared to children, resulting in subtler clinical manifestations. Therefore, beyond established risk factors such as dentures and chicken bone ingestion, increased clinical vigilance regarding FI is warranted in middle-aged and elderly populations.

### 3.3 Anatomic distribution of FIIP

Prior research has demonstrated that the anatomical distribution of foreign bodies varies according to object type. Fishbones are predominantly located in the tonsils (48.5%) and the base of the tongue (25.0%) (68). In these regions, symptoms typically manifest quickly and are often pronounced, prompting most patients to seek medical attention within 2 h (69). In our analysis of FIIP cases, the mean interval from ingestion or symptom onset to clinical presentation was 14.6 days. After excluding two statistical outliers (240 and 60 days), the adjusted mean presentation time was 8 days (Table 1). These observations indicate that the anatomical site of foreign body impaction correlates with the timing of presentation, the nature of the foreign body, and patient age. For fishbones, even when ingested intentionally, as in the case of our patient, the absence of a foreign body sensation in the oropharynx, tonsils, and esophagus is often perceived as safety. This perception significantly delays FI presentation, thereby increasing the incidence of FIIP.

#### 3.3.1 Small intestine

**Figure 7:**
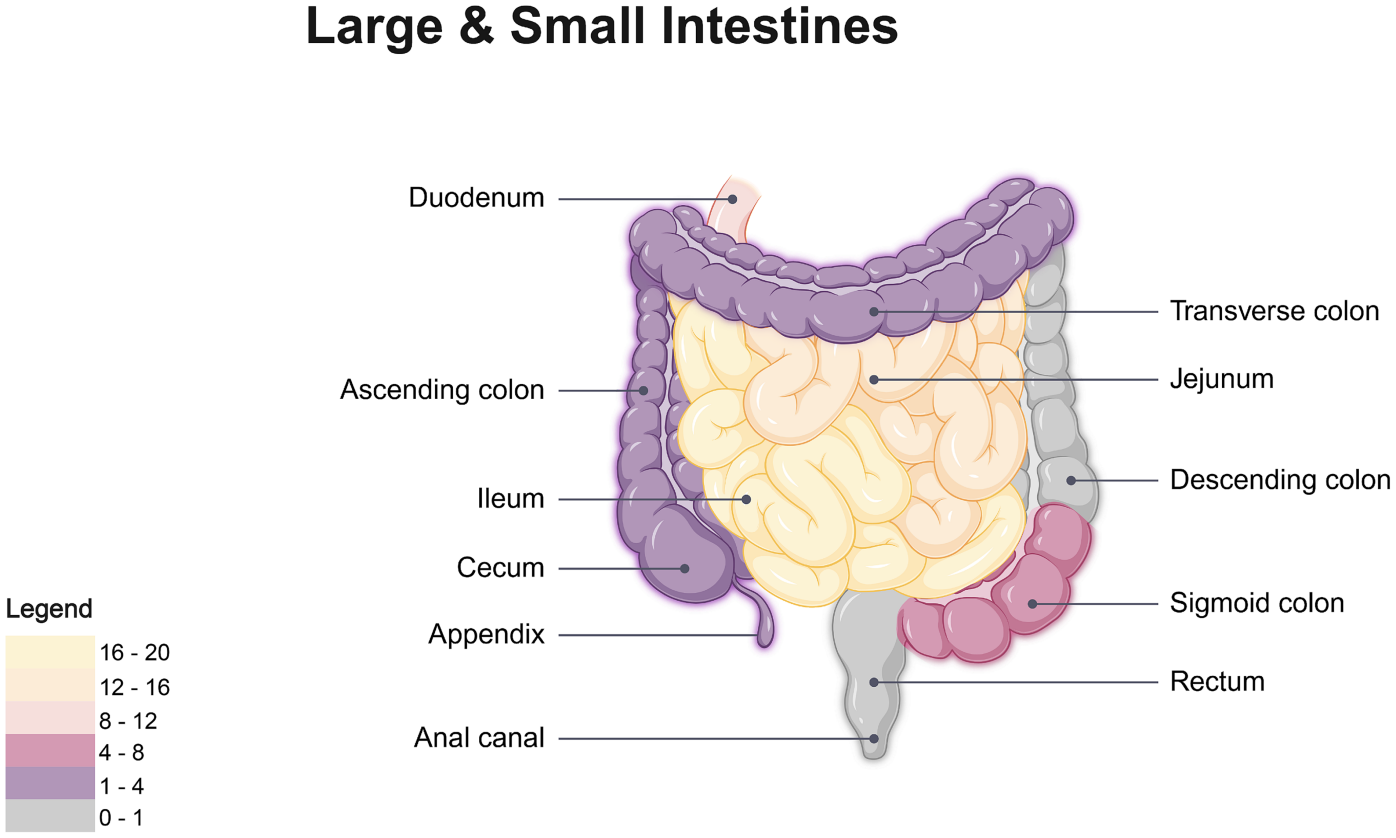
Location-based heatmap of FIIP frequency. Created with BioRender.com.

Anatomical configuration, luminal dimensions, and motility characteristics of the gastrointestinal tract influence the localization of FIIP, with the small intestine being the most frequently affected segment, accounting for 75.9% of cases (Table 2 and Figure 7). The small intestine is a long, tubular structure approximately 6–7 meters in length with an internal diameter of 3–4 cm (70). Its narrow, tortuous lumen, high mobility, and variable loop positioning increase the likelihood of foreign body impaction. Acute angulations, a common occurrence (71), may alter fishbone orientation and increase stress on the intestinal wall, promoting perforation. Among small intestinal segments, the ileum is most frequently involved, accounting for 45.4% of fishbone-related perforations (Table 2 and Figure 7). Compared to the jejunum, the ileum has a smaller lumen, greater length, more densely packed loops, and less vigorous peristalsis, especially in the terminal ileum and at the ileocecal valve (72). These anatomical and functional characteristics contribute to an elevated risk of fishbone retention and subsequent perforation. Multiple studies have identified the distal gastrointestinal tract, especially the ileum, as a common site of perforation, with 182 patients (58.5%) exhibiting involvement at this location (73).

**Table 2:** Distribution characteristics of intestinal segment perforation caused by fishbones (*N* = 58).

Site of intestinal perforation	Subgroup	Patients (No.)	Subcategory proportion (%)	Overall proportion (%)
SI		44		75.9
	Duo	8	18.2	13.8
	Jej	11	25.0	19.0
	Ile	20	45.4	34.5
	Unknown	5	11.4	8.6
LI		14		24.1
	Cec	3	21.4	5.2
	CO (AC)	1	7.1	1.7
	CO (TC)	2	14.3	3.4
	CO (SC)	7	50.0	12.1
	Rectosigmoid	1	7.1	1.7

Subcategory proportion refers to the proportion of a specific subgroup within its immediate superior category. Overall proportion refers to the proportion of perforation in a specific intestinal segment in the overall intestinal perforations. SI, small intestine; Duo, duodenum; Jej, jejunum; Ile, ileum; LI, large intestine; App., appendix; Cec, cecum; CO, colon; AC, ascending colon; TC, transverse colon; SC, sigmoid colon.

In cases of FIIP, a distinct feature is the involvement of Meckel’s diverticulum (MD) of the ileum, reported in eight patients (18.2%, Table 1). MD is a congenital gastrointestinal anomaly characterized by a small sac that protrudes from the small intestine wall. It is usually asymptomatic but may occasionally result in clinical complications (74). The prevalence of MD is estimated at approximately 2.2%, with a higher occurrence in males than females (75). The primary complications associated with MD include gastrointestinal hemorrhage, intestinal obstruction, diverticulitis, intussusception, and ulceration, while perforation and volvulus are considered rare (76). Foreign body-induced perforation of MD is exceedingly uncommon. Anatomically, MD averages 2.9 cm in length and 1.9 cm in width and features a significantly narrower lumen compared to the adjacent small intestine. The impaction of a fishbone within MD increases the likelihood of localized perforation (77). While most ingested foreign bodies pass through the gastrointestinal tract without incident, the fishbones associated with MD perforation in our series measured approximately 2 cm in length (Table 1). It is hypothesized that, even when the length of a fishbone or other foreign body is sufficient to traverse the normal gastrointestinal tract, the presence of MD may increase the risk of perforation. Surgical resection is generally recommended for symptomatic MD (78). However, the management of asymptomatic MD remains controversial. The estimated lifetime risk of complications in individuals with MD is approximately 5–6%. Accumulating evidence supports prophylactic resection of incidentally discovered MD in the absence of complicating factors such as peritonitis, hemodynamic instability, or ascites (79). In clinical practice, clinicians should maintain a high index of suspicion for FBI in patients with asymptomatic diverticula.

#### 3.3.2 Large intestine

Previous studies indicate that the sigmoid colon and cecum in the large intestine are common sites for foreign body perforation (80). The tortuous configuration and angulated structure of the intestinal lumen, coupled with relatively thin intestinal walls (81), predispose sharp or elongated foreign bodies to impaction at anatomical flexures, particularly near the ileocecal valve and rectosigmoid junction. Although fishbone-induced sigmoid colon perforation is rarely reported (82), our analysis indicates that such perforations in the large intestine occur most frequently in the sigmoid colon (50%), followed by the cecum (21.4%) (Table 2 and Figure 7). This distribution is attributed to the sigmoid colon’s anatomical characteristics, including its narrow diameter, increased tortuosity (83), and persistent fecal loading relative to other colonic segments (84). As water is absorbed by the colonic mucosa (85), fecal material becomes firm, and the vigorous peristaltic activity of the sigmoid colon elevates intraluminal pressure, thereby increasing the risk of perforation. Situated in the right iliac fossa, the cecum and ascending colon resemble sac-like structures with a larger diameter than the descending colon, sigmoid colon, and rectum, yet are anatomically constrained by a narrow ileocecal valve (86), limiting the passage of foreign bodies. Once lodged in the cecum, fishbones are more likely to perforate the thinner posterior wall (87).

Penetration of the intestinal wall by fishbones frequently induces localized inflammatory encapsulation and fibrotic barrier formation (88). As a result, fishbones rarely achieve full transmural penetration; when complete penetration occurs, they may migrate to adjacent or distant anatomical sites. One rare but potentially fatal complication is hepatic abscess formation following migration of a fishbone to the liver (59). Multiple case reports have documented instances in which ingested fishbones perforated the duodenum and subsequently migrated to the liver, resulting in hepatic abscesses (46, 89). In rare cases, such migration has been associated with serious vascular complications, including portal vein thrombosis and fistula formation involving the inferior vena cava (20, 21).

### 3.4 Intervention methods of FIIP

**Figure 8:**
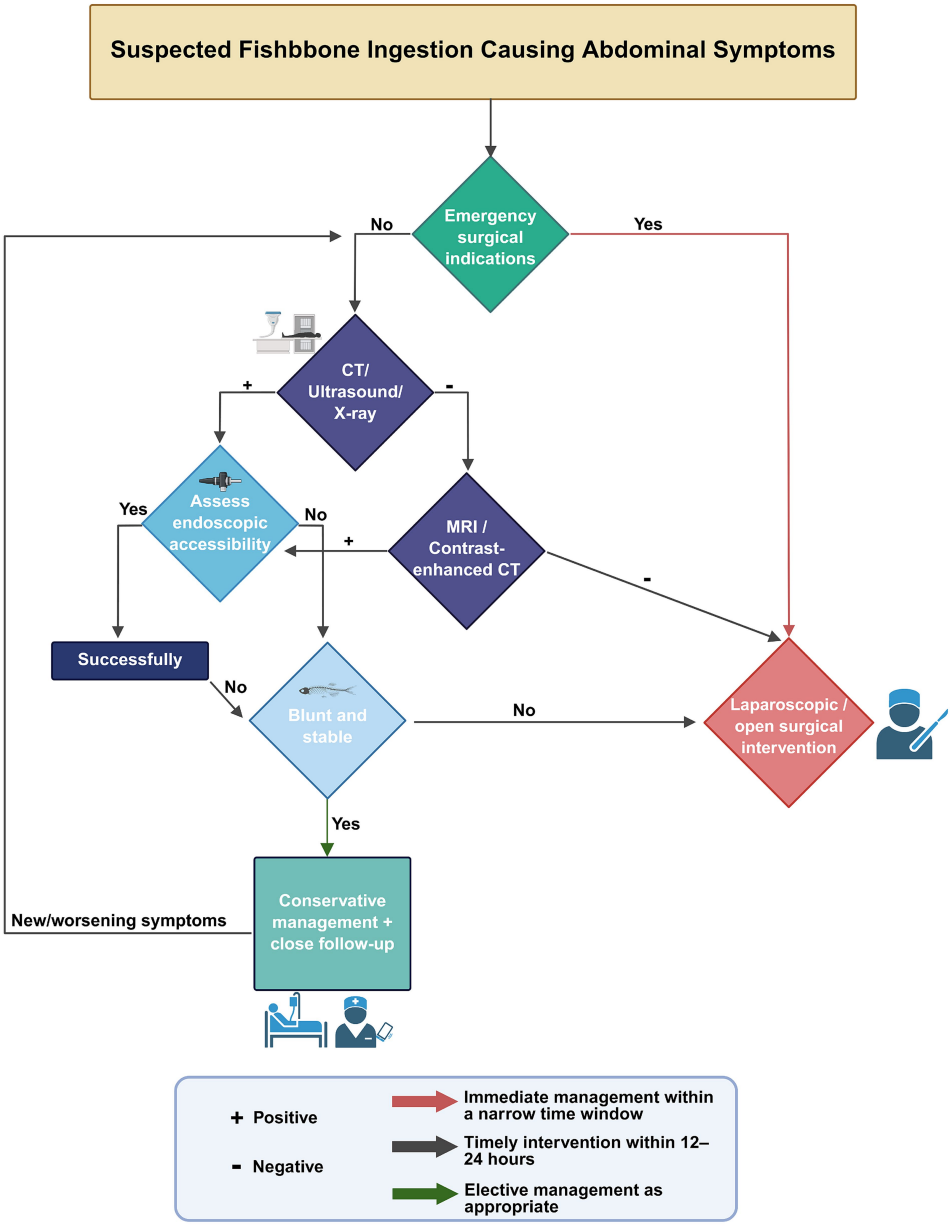
Clinical management algorithm for suspected fishbone ingestion presenting with abdominal symptoms. Created with BioRender.com.

FBI and food bolus impaction are commonly encountered in clinical practice. Over 80% of ingested foreign bodies pass through the gastrointestinal tract spontaneously, without requiring medical intervention. Endoscopic removal is indicated in approximately 20% of cases, whereas surgical intervention is required in fewer than 1% of patients (90). However, intestinal perforation secondary to FBI often necessitates surgical management (91). In our analysis of 58 cases of FIIP, 79.3% of patients underwent surgical treatment, 12.1% received endoscopic extraction, and 8.6% were managed conservatively (Table 3). When fish bone ingestion is suspected as the cause of abdominal symptoms, diagnostic and therapeutic procedures should be conducted in accordance with established clinical protocols (Figure 8).

**Table 3:** Distribution of interventions for intestinal perforation caused by fishbones (*N* = 58).

Intervention	Patients (No.)	Proportion (%)
Con Tx	5	8.6
Endoscopy	7	12.1
ST	46	79.3

Con Tx, conservative treatment; ST, surgical treatment.

#### 3.4.1 Conservative management

In cases with benign CT findings, such as the absence of peritoneal fluid, abscesses, pneumoperitoneum, fat stranding, or bowel wall thickening, or if these findings remain stable on serial CT scans, conservative management may be appropriate (92). Non-operative treatment of FIIP may also be appropriate for patients with stable vital signs, mild peritoneal signs, or contraindications to surgical intervention (93). For example, in the FIIP case reported by Ward et al. (94), only localized abdominal pain and mild leukocytosis with neutrophilia were observed, without other abnormal findings. The patient was managed with fasting alone, without antibiotics, and remained asymptomatic during 1 year of follow-up after discharge. In other conservatively treated cases, broad-spectrum antibiotics such as piperacillin-tazobactam or amoxicillin-clavulanate were administered in conjunction with bowel rest and supportive care (20, 23, 40). However, effective physician-patient communication is essential to ensure that patients are aware of the potential complications associated with conservative treatment. Close inpatient observation is necessary, and outpatient follow-up should be maintained for at least 1 year after discharge. Notably, there was a rare case of spontaneous expulsion of a 4.5-cm fishbone in an elderly patient with small bowel perforation, which occurred the day before the scheduled surgical intervention, nearly 2 months after ingestion (95). This unusual event may be explained by the thicker portion of the foreign body remaining lodged within the intestinal lumen, which eventually loosened and was expelled naturally.

Conservative management of FBI necessitates careful evaluation of potential complications (96). Foreign bodies lodged at perforation sites may irritate surrounding tissues, leading to chronic inflammation (97). Prolonged retention can result in the development of inflammatory granulomas, enteric fistulas, or intra-abdominal abscesses (98, 99). Elongated foreign bodies may also cause partial or complete intestinal obstruction, particularly in cases involving luminal stenosis, intussusception, or neoplastic lesions (100). Unstable perforation sites may exacerbate symptoms, as intestinal peristalsis can drive the object further into the bowel wall. If dislodged, the foreign body may perforate other intestinal segments, leading to peritonitis. Migration into adjacent organs—including the liver, bladder, uterus, ureter, abdominal wall, or blood vessels—can result in serious complications such as infection, hemorrhage, pseudoaneurysm formation, thrombosis, abscesses, fistulae, or ectopic inflammatory responses (101).

Given these risks, conservative treatment should be pursued cautiously and only under close surveillance. This is particularly important in cases where asymptomatic fish bones are incidentally identified on imaging but remain *in situ*. For such cases, treatment decisions should be highly individualized. Management should be based on the morphology of the foreign body (e.g., sharpness, length), its anatomical location, and the patient’s comorbidities. Blunt, short, and well-encapsulated fish bones may be managed conservatively, provided that patients undergo structured imaging surveillance, such as repeating CT every 2–3 days initially, then weekly, until the foreign body is expelled or becomes encapsulated and stabilized within the surrounding tissue. In contrast, sharp, long, or poorly fixed fish bones carry a higher risk of delayed perforation or migration. In elderly patients or those with immunosuppression or prior abdominal surgery, clinicians should maintain a lower threshold for early removal, even in the absence of symptoms.

Anatomical location is a crucial factor in guiding management decisions. Foreign bodies located near angulated or narrow segments (e.g., the ileocecal junction or sigmoid colon) present a higher risk of impaction or perforation, and may require earlier intervention. In contrast, asymptomatic fish bones that are stable on imaging and retained in the stomach or rectum are generally accessible and can usually be safely removed via endoscopy. If endoscopic removal is not feasible due to location or technical limitations, surgical consultation should be considered.

For patients with severe comorbidities or poor surgical candidacy, conservative management becomes even more critical. In these cases, sharp foreign bodies retained *in situ* should be monitored closely with regular imaging. The goal is to prevent delayed perforation or migration, which could worsen the patient’s condition. For patients in whom endoscopic removal is not feasible due to the location of the foreign body, conservative management becomes paramount. Alternative strategies, such as long-term observation and imaging follow-up, should be considered. In the absence of surgical options, the primary focus should be on minimizing further harm, managing symptoms, and preventing complications through vigilant monitoring.

Overall, conservative management of retained fish bones should be carefully selected based on patient factors and the nature of the foreign body, closely monitored, and promptly escalated if any signs of clinical deterioration or complication arise.

#### 3.4.2 Endoscopic treatment

**Figure 9:**
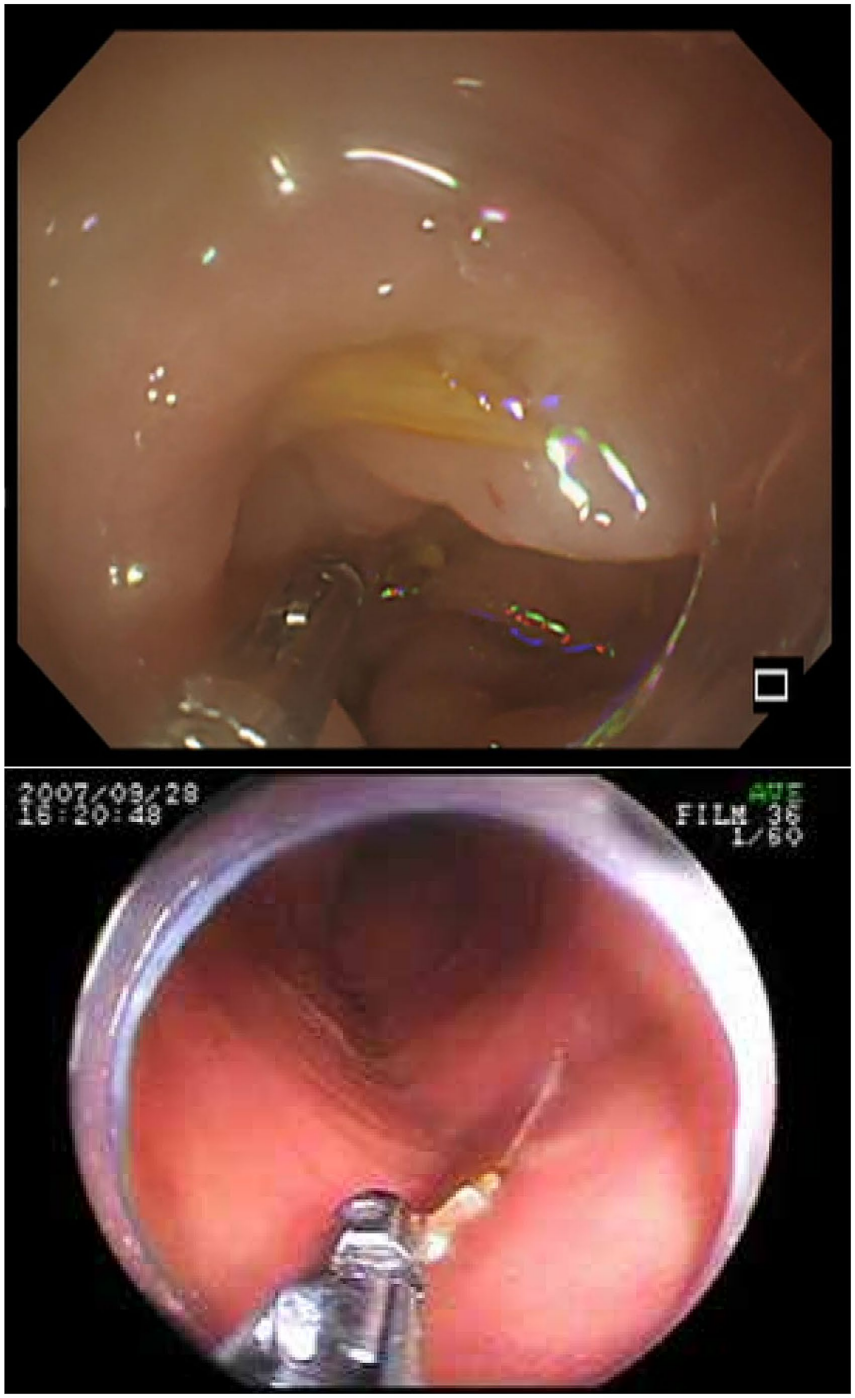
The figure shows the endoscopic findings of intestinal perforation caused by a fishbone. Top image: Endoscopic findings of edematous sigmoid colon mucosa. The fishbone is hidden within the edematous sigmoid colon. Adapted from reference (82) with permission from J-STAGE. Bottom image: The endoscopic image shows a tiny, sharp object lodged in the wall of the distal jejunum. The object was grasped with forceps and pulled out. Adapted from reference (52) with permission from International Scientific Information, Inc.

Endoscopic intervention is considered necessary in approximately 10–20% of FBI cases (102). Esophagogastroduodenoscopy is widely employed for the removal of upper gastrointestinal foreign bodies due to its safety, cost-effectiveness, procedural efficiency, and low complication rate (103). Therefore, in cases of duodenal perforation caused by FI, upper gastrointestinal endoscopy may be indicated for patients with mild symptoms and hemodynamic stability (51, 104) (Figure 9). However, reports of endoscopic management for lower gastrointestinal tract perforations remain limited (82). The advent of double-balloon enteroscopy has facilitated complete small intestinal examination and retrieval of retained foreign bodies, although the technique remains technically demanding (105–107). In two documented cases of fishbone-induced jejunal perforation, removal was successfully achieved using double-balloon enteroscopy with a snare, and both patients experienced no postoperative complications with rapid recovery. For sigmoid colon perforations, sigmoidoscopy equipped with a transparent cap enabled direct visualization and successful foreign body extraction (82, 108, 109). Whether suturing is required at the site of perforation remains unclear. In the seven cases of FIIP managed by endoscopic removal collected in this review (51–53, 82, 104, 108, 109), none reported the use of suturing. This may be attributed to the sharp and slender nature of fishbones, which typically result in small perforations that can be sealed by the surrounding inflammatory response, even without sutures. Most endoscopically treated cases did not present with peritonitis, and leakage of intestinal contents was rarely observed (104). In contrast to the others, one case documented the application of a clamp at the perforation site following endoscopic removal. Possibly due to its specific anatomical location. The sigmoid colon contains relatively dry contents compared to other intestinal segments (110), which may reduce the risk of intraperitoneal contamination. When perforation occurs in other segments of the intestine, even if the defect is small or encapsulated and the patient remains clinically stable, the risk of peritonitis due to leakage of liquid intestinal contents after endoscopic removal must still be carefully considered (111). At this stage, the decision to pursue endoscopic management or to suture the perforation site should be evaluated cautiously. Patients presenting with localized abdominal symptoms and stable systemic status may be candidates for conservative treatment using endoscopic techniques. Importantly, conversion from endoscopic removal to surgical intervention necessitates close multidisciplinary coordination and must be performed with heightened vigilance.

#### 3.4.3 Surgical treatment

Unlike most other cases of FBI, which seldom necessitate surgical intervention, the majority of FIIP cases in our cohort (78.9%) required operative management. In instances where patients presented with severe acute abdominal pain, emergency surgery was performed without a definitive diagnosis and occasionally even before CT scan (50, 112). Although CT imaging has demonstrated high sensitivity and specificity in detecting perforations caused by ingested foreign bodies, it is often bypassed in life-threatening scenarios. Therefore, in patients presenting with severe abdominal pain and suspected of FBI, an emergency abdominal CT scan should be prioritized to guide management decisions. Exploratory laparotomy is frequently recommended in cases of acute abdominal pain, particularly when signs of peritonitis are present or the diagnosis remains uncertain (113). In conclusion, surgical intervention is typically required for cases where conservative management or endoscopic retrieval has failed, or when the fishbone poses significant risks, such as perforation, obstruction, or infection. Both open and laparoscopic approaches have demonstrated therapeutic efficacy; however, the selection of surgical modality should be based on multiple factors, including the anatomical location and dimensions of the foreign body, the patient’s physiological status, and the extent of associated complications.

Laparotomy has traditionally been considered the gold standard for the management of gastrointestinal foreign body removal. It offers optimal visualization, operative access, and comprehensive exposure of the abdominal cavity. This approach is particularly appropriate in cases where the fishbone has migrated or caused substantial injury to adjacent structures, such as extensive abscess formation, diffuse peritonitis, hemorrhage, or bowel necrosis, all of which demand prompt surgical intervention. Laparotomy is also indicated for patients with a history of multiple laparotomies, dense intra-abdominal adhesions, or in elderly and frail individuals with compromised physiological reserves. Nevertheless, this method is associated with larger incisions, heightened postoperative discomfort, prolonged hospitalization, and an elevated risk of complications, including surgical site infection and adhesion formation.

Conversely, laparoscopic surgery has become increasingly favored in recent years owing to its minimally invasive characteristics. It is associated with smaller incisions, reduced postoperative discomfort, expedited recovery, shorter hospitalizations, and decreased rates of adhesion and surgical site infections (114). This technique is particularly advantageous in younger patients. When the fishbone is situated in an accessible anatomical location and the pathology is limited to a localized perforation, abscess, or solitary lesion, laparoscopy provides a safe and effective therapeutic option. However, this approach may not be appropriate for all patients, especially those with complex or deeply embedded foreign bodies, extensive intra-abdominal adhesions, or disseminated infections. In such scenarios, laparoscopic exploration may result in diagnostic inaccuracy, prolonged operative time, and potential iatrogenic complications (115). For the safety of the patient, laparoscopic exploration may be initially attempted during the early stages of disease or when the diagnosis remains uncertain, with conversion to laparotomy considered if necessary. Alternatively, a combined approach involving laparoscopy and mini-laparotomy may be employed to facilitate direct visualization and manual palpation, enabling thorough assessment of the perforation site (116). In our reported case, initial laparoscopic exploration was performed, but due to significant small bowel dilation and dense adhesions, the fishbone and the site of intestinal wall damage could not be identified, necessitating conversion to open surgery. For patients in whom laparoscopic findings are inconclusive, proceeding to direct laparotomy is recommended (117). During surgery, if intestinal necrosis is identified, resection of the affected bowel segment, primary anastomosis, or stoma creation may be warranted (14, 29, 118).

The increasing adoption of robotic surgery has demonstrated advantages such as enhanced visualization of the surgical field and improved instrument dexterity compared to both open and laparoscopic approaches, while also contributing to shorter hospital stays and a reduced risk of conversion to open surgery (119). Although robotic-assisted procedures have traditionally been reserved for elective surgeries (120), their application in emergency scenarios has been increasingly reported in recent years (121). As costs continue to decline, the integration of robotic surgery into emergency interventions for gastrointestinal perforation caused by foreign body ingestion is expected to expand.

### 3.5 Prognosis of FIIP

Most ingested foreign bodies do not lead to serious complications, and fatalities remain exceedingly rare (122). Among the reported cases, mortality related to foreign body aspiration is the most common cause of death (123). In the documented literature on fishbone-induced gastrointestinal perforation, only a single fatality has been reported, while the remaining patients experienced favorable clinical outcomes (Table 1). The deceased patient had morbid obesity and more than 16 underlying comorbidities. Additionally, delayed medical intervention—defined as a foreign body ingestion interval exceeding 15 days with the onset of severe symptoms—greatly impeded timely diagnosis and treatment. Despite surgical intervention, the patient ultimately succumbed to multiple organ dysfunction syndrome (124). In contrast, when diagnosis and treatment are performed promptly and comorbidities are minimal, the prognosis for fishbone-induced gastrointestinal perforation is generally favorable.

### 3.6 Fishbone length of FIIP

Prior studies have mainly emphasized the challenges associated with the size of ingested foreign bodies, particularly regarding their potential to cause gastrointestinal obstruction. Duodenal passage is determined by both the length and diameter of the foreign object, with items exceeding 6 cm in length or 2.5 cm in diameter being significantly less likely to pass through (1). In our cohort of FIIP cases, the fishbone lengths ranged from 1.1 to 5.1 cm, with a mean length of 3.0 cm. The largest dimension recorded was a fish fin measuring 4.0 cm × 3.0 cm (Table 1). These measurements are comparable to the average diameter of the small intestine. Fishbones exceeding the intestinal lumen’s diameter may increase the risk of small bowel perforation. Therefore, in cases of FBI, both the size and physical characteristics—such as sharpness and rigidity—should be carefully evaluated. The mechanism of injury differs markedly between blunt and sharp foreign bodies. Notably, perforation risk is primarily determined by the shape of sharp objects; even a length of 1 cm may be sufficient to penetrate the full thickness of the bowel wall.

### 3.7 Fish species of FIIP

**Figure 10:**
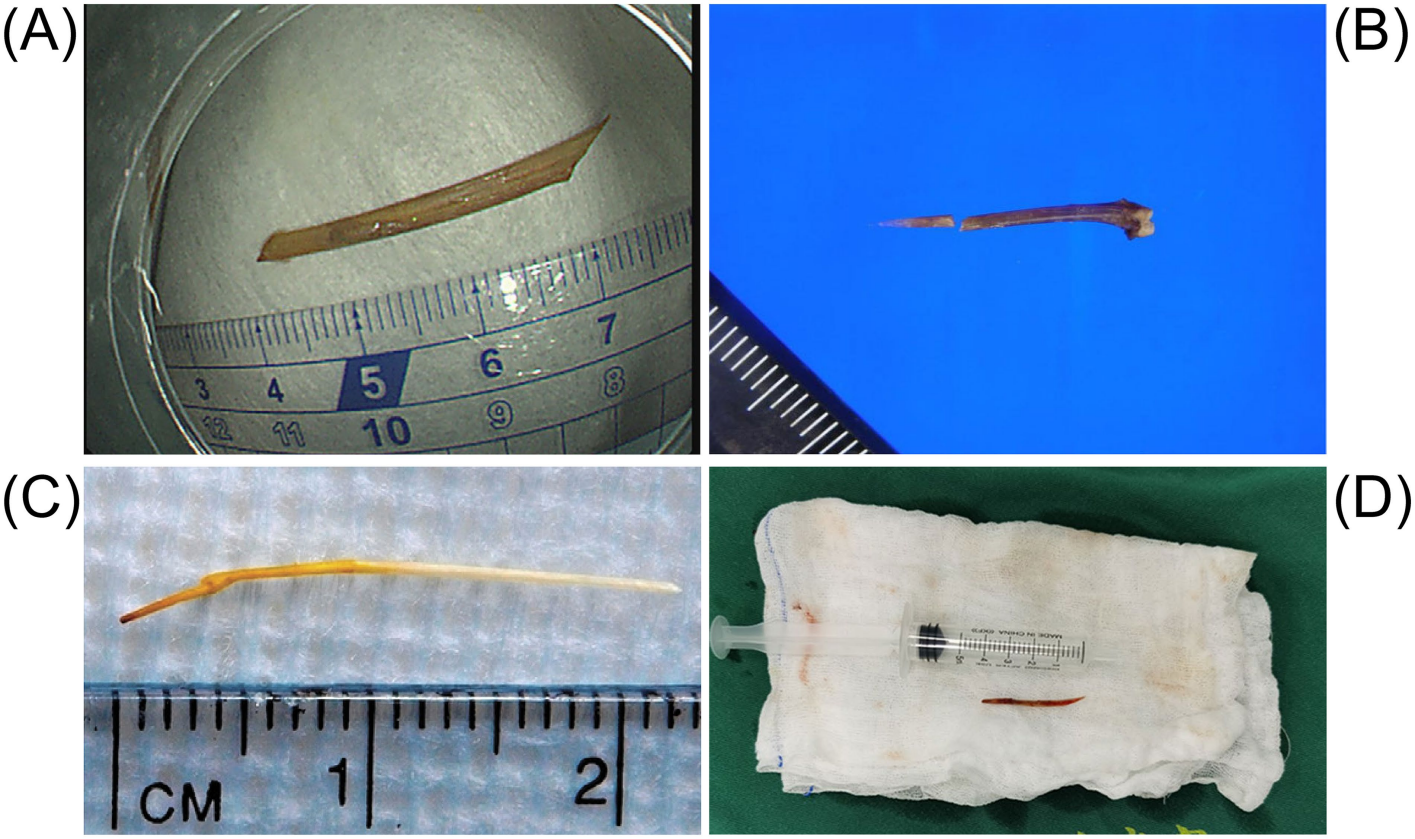
The figure illustrates the shape and length of the fishbone that caused the intestinal perforation. **(A)** The image shows the fishbone removed by endoscopic examination. Adapted from reference (82) with permission from J-STAGE. **(B)** The object is a 2-centimeter-long fishbone that was removed from the cecal wall. Adapted from reference (19) with permission from Springer Nature. **(C)** The object is a carp fishbone that caused the perforation of the jejunum. Adapted from reference (53) with permission from Elsevier. **(D)** The fishbone retrieved from the abdominal cavity resembles a knife. Adapted from reference (41) with permission from Springer Nature.

In most previously reported cases of intestinal perforation caused by fishbones, the specific fish species involved were not identified. Among the documented cases, only 14 mentioned the species. The implicated fishbones were typically slender and pointed (Figure 10). Both the fish species and cooking methods may influence bone morphology, density, and resistance to gastric acid dissolution (125). These factors play a key role in determining the site of impaction and the likelihood of perforation (126). Regional dietary preferences influence the types of fish consumed, potentially explaining geographic variations in incidence (127). Clinically, it is advisable to document the fish species whenever possible, as this may assist in predicting the location, size, and risk associated with the ingested bone, thus improving diagnostic and therapeutic accuracy. Future case reports are encouraged to include such information to enhance clinical understanding and global data sharing.

### 3.8 Regional characteristics of FIIP

**Figure 11:**
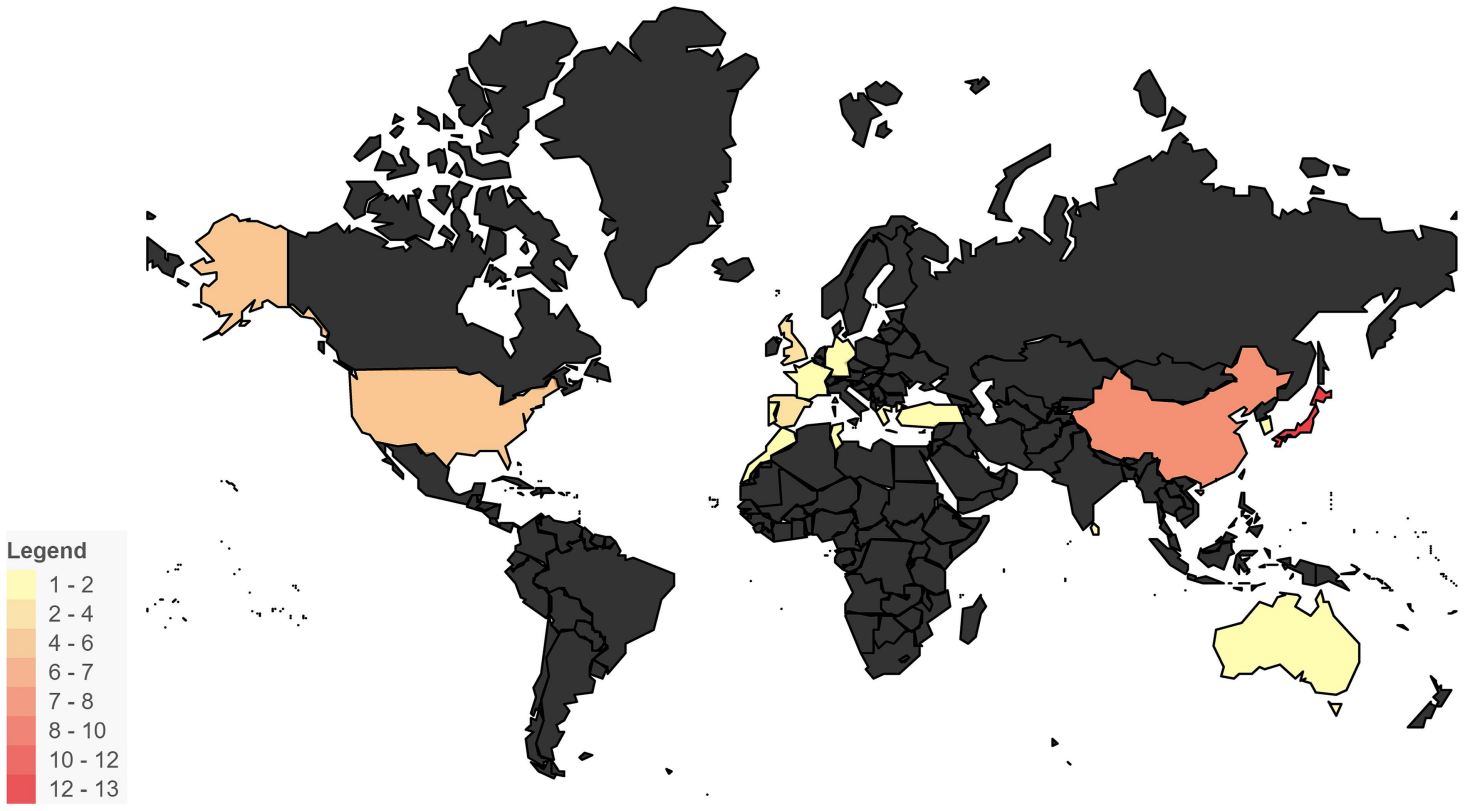
Geographical distribution of FIIP cases. This heatmap illustrates the geographical distribution of FIIP cases, highlighting regions with a higher number of occurrences.

Fish are abundant in bioactive compounds possessing immunomodulatory, antioxidant, antimicrobial, neuroprotective, and cardioprotective properties (128). Regions with extensive coastlines and rich inland water resources hold substantial potential for aquaculture development (129), in recent years, both global fish consumption and production have steadily increased (130). Asia remains the leading producer in the aquaculture sector, contributing 92% of global output (131), and records the highest per capita fish consumption worldwide (132). This heavy dietary reliance significantly increases the risk of fishbone-related injuries. In our dataset of FIIP cases, 63.8% involved patients from Asia (Figure 11 and Table 1). China stands as the world’s largest producer and consumer of aquatic products (133), supported by its expansive coastline and abundant freshwater resources. In addition to seafood, freshwater fish species such as carp and catfish, which are characterized by numerous sharp bones, are widely consumed, particularly in Asia (134). In our collected cases, 12.1% originated from mainland China and Taiwan, China (Figure 11 and Table 1). Japan accounted for the highest proportion of reported cases globally, at 22.4% (Figure 11 and Table 1). This elevated incidence in Japan may be related to several factors: a traditionally higher consumption of fish compared to meat until 2007 (135), a rapidly aging population with a high dietary intake of fish (136), and cultural preferences for raw fish and fried fishbones (137). Furthermore, underreporting in rural regions—due to limited access to medical facilities—may exacerbate the true burden across Asia (138). Overall, regional dietary customs, high fish consumption driven by aquaculture, and unequal healthcare access collectively contribute to the increased incidence of fishbone-related intestinal perforation across Asia, especially in East Asia.

## 4 Conclusion

FBI remains a prominent clinical concern in emergency medicine. The systematic collection of epidemiological and clinical data on FBI is vital for enhancing diagnostic precision and informing effective treatment strategies. Currently, data on intestinal perforation due to FIIP remain limited. Our study is inherently limited by its retrospective nature and small sample size. However, it provides the first comprehensive analysis of FIIP as a distinct subset of gastrointestinal foreign body injuries. It addresses key aspects, including thorough clinical history-taking, the need for greater awareness of FI risks in elderly populations, identification of high-risk anatomical sites for FIIP, the pivotal role of CT in diagnosis, treatment selection, regional clustering trends, and dietary risk factors linked to fish consumption. We aim for this report to serve as a meaningful reference for advancing both clinical management and future research in this field.

## Glossary

FBI: Foreign body ingestion
FI: Fishbone ingestion
CT: Computed tomography
FIIP: Fishbone-induced intestinal perforation
WBC: White blood cell
NEUT: Neutrophil
CRP: C-reactive protein
MD: Meckel’s diverticulum
Con Tx: Conservative treatment
ST: Surgical treatment
LS: Laparoscopic surgery
Lap: Laparotomy
Abs: Antibiotics
SI: Small intestine
Duo: Duodenum
Jej: Jejunum
Ile: Ileum
LI: Large intestine
Cec: Cecum
CO: Colon
TC: Transverse colon
SC: Sigmoid colon
App.: Appendix
AC: Ascending colon

## References

[1] Tseng HJ Hanna TN Shuaib W Aized M Khosa F Linnau KF. Imaging foreign bodies: ingested, aspirated, and inserted. Ann Emerg Med. (2015) 66:570–582e5. doi: 10.1016/j.annemergmed.2015.07.499, PMID: 26320521

[2] Hunter TB Taljanovic MS. Foreign bodies. Radiographics. (2003) 23:731–57. doi: 10.1148/rg.233025137, PMID: 12740473

[3] Kehagias D Mulita F Maroulis I Benetatos N. Caudate lobe: the last barrier—an unusual place for a foreign body. ANZ J Surg. (2022) 92:1218–20. doi: 10.1111/ans.17226, PMID: 34550639

[4] Velitchkov NG Grigorov GI Losanoff JE Kjossev KT. Ingested foreign bodies of the gastrointestinal tract: retrospective analysis of 542 cases. World J Surg. (1996) 20:1001–5. doi: 10.1007/s002689900152, PMID: 8798356

[5] Pernar LIM Mongiu A Sheu EG. An unusual cause of intestinal perforation. JAMA Surg. (2017) 152:199. doi: 10.1001/jamasurg.2016.4614, PMID: 27926750

[6] Du H Guo Y Bennett DA Bragg F Bian Z Chadni M. Red meat, poultry and fish consumption and risk of diabetes: a 9 year prospective cohort study of the China Kadoorie Biobank. Diabetologia. (2020) 63:767–79. doi: 10.1007/s00125-020-05091-x 31970429 PMC7054352

[7] Kwasek K Thorne-Lyman AL Phillips M. Can human nutrition be improved through better fish feeding practices? A review paper. Crit Rev Food Sci Nutr. (2020) 60:3822–35. doi: 10.1080/10408398.2019.1708698, PMID: 31983214

[8] Sahn B Mamula P Ford CA. Review of foreign body ingestion and esophageal food impaction management in adolescents. J Adolesc Health. (2014) 55:260–6. doi: 10.1016/j.jadohealth.2014.01.022, PMID: 24686070

[9] Binder L Anderson WA. Pediatric gastrointestinal foreign body ingestions. Ann Emerg Med. (1984) 13:112–7. doi: 10.1016/S0196-0644(84)80573-9, PMID: 6691612

[10] Vizcarrondo FJ Brady PG Juergen NH. Foreign bodies of the upper gastrointestinal tract. Gastrointest Endosc. (1983) 29:208–10. doi: 10.1016/S0016-5107(83)72586-1, PMID: 6618118

[11] Zamary KR Davis JW Ament EE Dirks RC Garry JE. This too shall pass: a study of ingested sharp foreign bodies. J Trauma Acute Care Surg. (2017) 82:150–5. doi: 10.1097/TA.0000000000001265, PMID: 27805997

[12] Selivanov V Sheldon GF Cello JP Crass RA. Management of foreign body ingestion. Ann Surg. (1984) 199:187–91. doi: 10.1097/00000658-198402000-00010, PMID: 6696536 PMC1353331

[13] Schwartz JT Graham DY. Toothpick perforation of the intestines. Ann Surg. (1977) 185:64–6. doi: 10.1097/00000658-197701000-00010, PMID: 318821 PMC1396248

[14] Choi Y. Peritonitis with small bowel perforation caused by a fish bone in a healthy patient. World J Gastroenterol. (2014) 20:1626. doi: 10.3748/wjg.v20.i6.1626, PMID: 24587641 PMC3925874

[15] Chen CK Su YJ Lai YC Cheng HKH Chang WH. Fish bone-related intra-abdominal abscess in an elderly patient. Int J Infect Dis. (2010) 14:e171–2. doi: 10.1016/j.ijid.2009.03.024, PMID: 19541523

[16] Ambe P Weber SA Schauer M Knoefel WT. Swallowed foreign bodies in adults. Dtsch Arztebl Int. (2012) 109:869–75. doi: 10.3238/arztebl.2012.0869, PMID: 23293675 PMC3536040

[17] Hoevenaars FPM Berendsen CMM Pasman WJ van den Broek TJ Barrat E de Hoogh IM. Evaluation of food-intake behavior in a healthy population: personalized vs. one-size-fits-all. Nutrients. (2020) 12:2819. doi: 10.3390/nu12092819, PMID: 32942627 PMC7551874

[18] Oken E Choi AL Karagas MR Mariën K Rheinberger CM Schoeny R. Which fish should I eat? Perspectives influencing fish consumption choices. Environ Health Perspect. (2012) 120:790–8. doi: 10.1289/ehp.1104500, PMID: 22534056 PMC3385441

[19] Kuwahara K Mokuno Y Matsubara H Kaneko H Shamoto M Iyomasa S. Development of an abdominal wall abscess caused by fish bone ingestion: a case report. J Med Case Rep. (2019) 13:369. doi: 10.1186/s13256-019-2301-7, PMID: 31837708 PMC6911699

[20] Brandão D Canedo A Maia M Ferreira J Vaz G. Duodenocaval fistula as a result of a fish bone perforation. J Vasc Surg. (2010) 51:1276–8. doi: 10.1016/j.jvs.2009.12.049, PMID: 20223621

[21] Wang VY Wang VL Kao L Elwood DR. A complex game of go fish: a hybrid endoscopic and surgical approach to a fish bone perforation of the portal vein. Am Surg. (2020) 86:153–5. doi: 10.1177/000313482008600317 32223827

[22] Chiu WY Chen YJ Cheng PC Shiau EL. Early presentation of bowel perforation due to fish bone ingestion. QJM. (2014) 107:679–80. doi: 10.1093/qjmed/hcu004, PMID: 24440924

[23] Kuo CC. Medical treatment for a fish bone-induced ileal micro-perforation: a case report. World J Gastroenterol. (2012) 18:5994. doi: 10.3748/wjg.v18.i41.5994, PMID: 23139620 PMC3491611

[24] Bhatia R Deane AJB Landham P Schulte KM. An unusual case of bowel perforation due to fish fin ingestion: bowel perforation due to fish fin ingestion. Int J Clin Pract. (2006) 60:229–31. doi: 10.1111/j.1742-1241.2006.00610.x, PMID: 16451298

[25] Bathla G Teo LL Dhanda S. Pictorial essay: complications of a swallowed fish bone. Indian J Radiol Imaging. (2011) 21:63–8. doi: 10.4103/0971-3026.76061, PMID: 21431037 PMC3056375

[26] Goh BKP Jeyaraj PR Chan HS Ong HS Agasthian T Chang KTE. Case report: a case of fish bone perforation of the stomach mimicking a locally advanced pancreatic carcinoma. Dig Dis Sci. (2004) 49:1935–7. doi: 10.1007/s10620-004-9595-y, PMID: 15628728

[27] Paixão TSA Leão RV de Souza Maciel Rocha Horvat N Viana PCC Da Costa Leite C de Azambuja RL. Abdominal manifestations of fishbone perforation: a pictorial essay. Abdom Radiol. (2017) 42:1087–95. doi: 10.1007/s00261-016-0939-9, PMID: 27717979

[28] Saunders D Jones M Kaushik M Thomas WM. Fish bone perforation of the terminal ileum presenting as acute appendicitis. BMJ Case Rep. (2014) 2014:bcr2013009533. doi: 10.1136/bcr-2013-009533, PMID: 24639331 PMC3962970

[29] Hsu SD Chan DC Liu YC. Small-bowel perforation caused by fish bone. World J Gastroenterol. (2005) 11:1884–5. doi: 10.3748/wjg.v11.i12.1884, PMID: 15793887 PMC4305897

[30] Wong JH Suhaili DN Kok KY. Fish bone perforation of Meckel’s diverticulum: a rare event? Asian J Surg. (2005) 28:295–6. doi: 10.1016/s1015-9584(09)60364-x 16234083

[31] Mouawad NJ Hammond S Kaoutzanis C. Perforation of Meckel’s diverticulum by an intact fish bone. BMJ Case Rep. (2013) 2013:bcr2012008226. doi: 10.1136/bcr-2012-008226, PMID: 23429021 PMC3604403

[32] McDowell DE Bush M. Fish bone perforation of Meckel’s diverticulum simulating a leaking abdominal aortic aneurysm. South Med J. (1982) 75:891–2. doi: 10.1097/00007611-198207000-00037, PMID: 7089664

[33] Yogev D Mahameed F Gileles-Hillel A Millman P Davidovics Z Hashavya S. Hijab pin ingestions. Pediatrics. (2020) 145:e20193472. doi: 10.1542/peds.2019-3472, PMID: 32385133

[34] Alansari AN Baykuziyev T Soyer T Akıncı SM Al Ali KK Aljneibi A. Magnet ingestion in growing children: a multi-center observational study on single and multiple magnet incidents. Sci Rep. (2024) 14:4575. doi: 10.1038/s41598-024-55127-0, PMID: 38403623 PMC10894856

[35] Mubarak A Benninga MA Broekaert I Dolinsek J Homan M Mas E. Diagnosis, management, and prevention of button battery ingestion in childhood: a European Society for Paediatric Gastroenterology Hepatology and Nutrition position paper. J Pediatr Gastroenterol Nutr. (2021) 73:129–36. doi: 10.1097/MPG.0000000000003048, PMID: 33555169

[36] Kijowski R Demehri S Roemer F Guermazi A. Osteoarthritis year in review 2019: imaging. Osteoarthr Cartil. (2020) 28:285–95. doi: 10.1016/j.joca.2019.11.009, PMID: 31877380

[37] Hillman KM. Pneumoperitoneum—a review. Crit Care Med. (1982) 10:476–81. doi: 10.1097/00003246-198207000-00015, PMID: 7044684

[38] Berk RN Reit RJ. Intra-abdominal chicken-bone abscess. Radiology. (1971) 101:311–3. doi: 10.1148/101.2.311, PMID: 5114772

[39] Kumar M Joseph G Kumar S Clayton M. Fish bone as a foreign body. J Laryngol Otol. (2003) 117:568–9. doi: 10.1258/002221503322113058 12901817

[40] Mora-Guzmán I Valdés De Anca Á Martín-Pérez E. Intra-abdominal abscess due to fish bone perforation of small bowel. Acta Chir Belg. (2019) 119:66–7. doi: 10.1080/00015458.2018.1493822, PMID: 30010485

[41] Wang R He J Chen Z Wen K. Migration of fish bones into abdominal para-aortic tissue from the duodenum after leading to duodenal perforation: a case report. BMC Gastroenterol. (2021) 21:82. doi: 10.1186/s12876-021-01662-3, PMID: 33622248 PMC7903620

[42] Natsuki S Iseki Y Nagahara H Fukuoka T Shibutani M Ohira M. Liver abscess caused by fish bone perforation of Meckel’s diverticulum: a case report. BMC Surg. (2020) 20:121. doi: 10.1186/s12893-020-00783-y, PMID: 32503493 PMC7275410

[43] Ngan JH Fok PJ Lai EC Branicki FJ Wong J. A prospective study on fish bone ingestion. Experience of 358 patients. Ann Surg. (1990) 211:459–62. doi: 10.1097/00000658-199004000-00012, PMID: 2322040 PMC1358032

[44] Kessner R Barnes S Halpern P Makrin V Blachar A. CT for acute nontraumatic abdominal pain-is oral contrast really required? Acad Radiol. (2017) 24:840–5. doi: 10.1016/j.acra.2017.01.013, PMID: 28237189

[45] Goh BKP Tan YM Lin SE Chow PKH Cheah FK Ooi LLPJ. CT in the preoperative diagnosis of fish bone perforation of the gastrointestinal tract. AJR Am J Roentgenol. (2006) 187:710–4. doi: 10.2214/AJR.05.0178, PMID: 16928935

[46] Goh BKP Yong WS Yeo AWY. Pancreatic and hepatic abscess secondary to fish bone perforation of the duodenum. Dig Dis Sci. (2005) 50:1103–6. doi: 10.1007/s10620-005-2712-8, PMID: 15986862

[47] Song J Yang W Zhu Y Fang Y Qiu J Qiu J. Ingested a fish bone-induced ileal perforation: a case report. Medicine. (2020) 99:e19508. doi: 10.1097/MD.0000000000019508, PMID: 32282701 PMC7440113

[48] Lin CY Wu FZ. Fish bone perforation of small intestine. QJM. (2012) 105:479–80. doi: 10.1093/qjmed/hcr035 21382926

[49] Lunsford KE Sudan R. Small bowel perforation by a clinically unsuspected fish bone: laparoscopic treatment and review of literature. J Gastrointest Surg. (2012) 16:218–22. doi: 10.1007/s11605-011-1610-y, PMID: 21796463

[50] Drakonaki E Chatzioannou M Spiridakis K Panagiotakis G. Acute abdomen caused by a small bowel perforation due to a clinically unsuspected fish bone. Diagn Interv Radiol. (2011) 17:160–2. doi: 10.4261/1305-3825.DIR.3236-09.1, PMID: 20683816

[51] Völk M Geissler A Herold T Schäffler A Gross V Gmeinwieser J. The computed tomographic demonstration of duodenal perforation caused by a fish bone. Fortschr Geb Rontgenstr Nuklearmed. (1997) 167:198–200. 10.1055/s-2007-1015516 9333363

[52] Shibuya T Osada T Asaoka D Mori H Beppu K Sakamoto N. Double-balloon endoscopy for treatment of long-term abdominal discomfort due to small bowel penetration by an eel bone. Med Sci Monit. (2008) 14:CS107-9 18830197

[53] Alkhatib AA Umar SB Patel NC Harrison ME. Balloon-assisted enteroscopy for the treatment of a sealed jejunal perforation: removal of a penetrating fish bone (with video). Gastrointest Endosc. (2013) 77:133–5. doi: 10.1016/j.gie.2012.08.033, PMID: 23062761

[54] Matricardi L Lovati R. Intestinal perforation by a foreign body: diagnostic usefulness of ultrasonography. J Clin Ultrasound. (1992) 20:194–6. doi: 10.1002/jcu.1870200306, PMID: 1313833

[55] White RZ Rezaian P Parasuramar A Sampson MJ. Ultrasound-assisted foreign body extraction (U-SAFE): review of technique and technical pearls. J Med Imaging Radiat Oncol. (2022) 66:362–9. doi: 10.1111/1754-9485.13313, PMID: 34396705

[56] Rooks VJ Shiels WE Murakami JW. Soft tissue foreign bodies: a training manual for sonographic diagnosis and guided removal. J Clin Ultrasound. (2020) 48:330–6. doi: 10.1002/jcu.22856, PMID: 32385865 PMC7754500

[57] Athreya S Radhachandran A Ivezić V Sant VR Arnold CW Speier W. Enhancing ultrasound image quality across disease domains: application of cycle-consistent generative adversarial network and perceptual loss. JMIR Biomed Eng. (2024) 9:e58911. doi: 10.2196/58911, PMID: 39689310 PMC11688586

[58] Masuya R Okamoto K Kidogawa H Kamizono J Ieiri S. Rare pediatric case of Meckel diverticulum penetration caused by a fish bone. Pediatr Int. (2019) 61:731–3. doi: 10.1111/ped.13902, PMID: 31309652

[59] Yen H Hsu Y. Gastrointestinal: pyogenic liver abscess associated with a penetrating fish bone. J Gastroenterol Hepatol. (2010) 25:1900–21. doi: 10.1111/j.1440-1746.2010.06551.x, PMID: 21155186

[60] Gonçalves A Almeida M Malheiro L Costa-Maia J. Meckel’s diverticulum perforation by a fish bone: a case report. Int J Surg Case Rep. (2016) 28:237–40. doi: 10.1016/j.ijscr.2016.08.026, PMID: 27744259 PMC5066196

[61] Pinto A Pinto F Faggian A Rubini G Caranci F Macarini L. Sources of error in emergency ultrasonography. Crit Ultrasound J. (2013) 5:S1. doi: 10.1186/2036-7902-5-S1-S1 23902656 PMC3711733

[62] Zhong Z Li Z Xing Y Guo S. Case report: a large gastric calcifying fibrous tumor treated with endoscopic submucosal excavation. Front Oncol. (2024) 14:1385695. doi: 10.3389/fonc.2024.1385695, PMID: 39188678 PMC11345178

[63] Mantegazza C Destro F Ferraro S Biganzoli D Marano G Quitadamo P. Recent trends in foreign body ingestion (FBI) epidemiology: a national cohort study. Dig Liver Dis. (2025) 57:595–602. doi: 10.1016/j.dld.2024.10.002, PMID: 39477708

[64] Oliva S Romano C de Angelis P Isoldi S Mantegazza C Felici E. Foreign body and caustic ingestions in children: a clinical practice guideline. Dig Liver Dis. (2020) 52:1266–81. doi: 10.1016/j.dld.2020.07.016, PMID: 32782094

[65] van Waas M De Bruyne P de Ridder L. Paediatric multiple magnet ingestion. Lancet Gastroenterol Hepatol. (2021) 6:80. doi: 10.1016/S2468-1253(20)30338-1 33308436

[66] Orsagh-Yentis D McAdams RJ Roberts KJ McKenzie LB. Foreign-body ingestions of young children treated in US emergency departments: 1995–2015. Pediatrics. (2019) 143:e20181988. doi: 10.1542/peds.2018-1988, PMID: 30979810

[67] Marquardt P Derousseau T Patel N. Foreign body ingestion: a curious case of the missing denture. Geriatrics. (2020) 5:49. doi: 10.3390/geriatrics5030049, PMID: 32933211 PMC7555874

[68] Kim SY Park B Kong IG Choi HG. Analysis of ingested foreign bodies according to age, type and location: a retrospective observational study. Clin Otolaryngol. (2016) 41:640–5. doi: 10.1111/coa.12576, PMID: 26505266

[69] Wu T Meng J Li X He S. A rare and peculiar case of fish bone penetration to the submucosa of the posterior pharyngeal wall. Asian J Surg. (2024) 47:1445–6. doi: 10.1016/j.asjsur.2023.11.123 38072702

[70] Jung SM Kim S. *In vitro* models of the small intestine for studying intestinal diseases. Front Microbiol. (2021) 12:767038. doi: 10.3389/fmicb.2021.767038, PMID: 35058894 PMC8765704

[71] Browning CM Cloutier R Rich TC Leavesley SJ. Endoscopy lifetime systems architecture: scoping out the past to diagnose the future technology. Systems. (2022) 10:189. doi: 10.3390/systems10050189, PMID: 36330206 PMC9627979

[72] Pichardo J Zapata J Echavarría R Ubiñas R Báez P Gómez Á. Gallstone ileus with cholecystoenteric fistula in an elderly female: a case report. Cureus. (2023) 15:e37077. doi: 10.7759/cureus.37077, PMID: 37153256 PMC10156418

[73] Memon AA Siddiqui FG Abro AH Agha AH Lubna S Memon AS. An audit of secondary peritonitis at a tertiary care university hospital of Sindh, Pakistan. World J Emerg Surg. (2012) 7:6. doi: 10.1186/1749-7922-7-6, PMID: 22423629 PMC3319418

[74] Ivatury RR. Meckel’s diverticulum and the eponymous legend. J Trauma Acute Care Surg. (2019) 87:451–5. doi: 10.1097/TA.0000000000002300, PMID: 31349351

[75] Levy AD Hobbs CM. From the archives of the AFIP. Meckel diverticulum: radiologic features with pathologic correlation. Radiographics. (2004) 24:565–87. doi: 10.1148/rg.242035187, PMID: 15026601

[76] Sagar J Kumar V Shah DK. Meckel’s diverticulum: a systematic review. J R Soc Med. (2006) 99:501–5. doi: 10.1177/014107680609901011 17021300 PMC1592061

[77] Uppal K Tubbs RS Matusz P Shaffer K Loukas M. Meckel’s diverticulum: a review. Clin Anat. (2011) 24:416–22. doi: 10.1002/ca.21094, PMID: 21322060

[78] Ding Y Zhou Y Ji Z Zhang J Wang Q. Laparoscopic management of perforated Meckel’s diverticulum in adults. Int J Med Sci. (2012) 9:243–7. doi: 10.7150/ijms.4170, PMID: 22577339 PMC3348529

[79] Malik AA Shams-ul-Bari Wani KA Khaja AR. Meckel’s diverticulum-revisited. Saudi J Gastroenterol. (2010) 16:3–7. doi: 10.4103/1319-3767.58760 20065566 PMC3023098

[80] Devanathan N Patel H Sargin P Zarak A Biglione A. Sigmoid perforation by an ingested foreign body mimicking acute appendicitis: a case report. Cureus. (2024) 16:e66855. doi: 10.7759/cureus.66855, PMID: 39280396 PMC11396609

[81] Lu J Tan Y Liu D Li C Zhou H. Endoscopic submucosal dissection for rectal-sigmoid laterally spreading tumors ≥10 cm: an analysis of 10 cases. Transl Cancer Res. (2021) 10:867–75. doi: 10.21037/tcr-20-2659, PMID: 35116416 PMC8798390

[82] Ueda T Sato H Ogimi T Deguchi R Suzuki H. Use of endoscopy to remove fish bone that caused sigmoid colon perforation. Intern Med. (2024) 63:2626–30. doi: 10.2169/internalmedicine.3063-23, PMID: 38369354 PMC11518597

[83] Manes G Andreozzi P Omazzi B Bezzio C Redaelli D Devani M. Efficacy of withdrawal time monitoring in adenoma detection with or without the aid of a full-spectrum scope. Endosc Int Open. (2019) 7:E1135–42. doi: 10.1055/a-0854-3946 31475231 PMC6715423

[84] Paulus LP Buehler A Wagner AL Raming R Jüngert J Simon D. Contrast-enhanced multispectral optoacoustic tomography for functional assessment of the gastrointestinal tract. Adv Sci. (2023) 10:e2302562. doi: 10.1002/advs.202302562, PMID: 37289088 PMC10427354

[85] Guo L Zhang T Liang T Chen J Gao H. Laparoscopic radical cystectomy with ileal orthotopic neobladder for bladder cancer: current indications and outcomes. Urol Int. (2024) 108:242–53. doi: 10.1159/000535032, PMID: 37995673 PMC11151991

[86] Hussain MS Balagoni H Dwivedi S Piper M. Macroscopic to microscopic—a case of Crohn’s disease progressing to collagenous colitis. Cureus. (2021) 13:e18299. doi: 10.7759/cureus.18299 34722074 PMC8547377

[87] Pérez-Lara JL Santana Y Hernández-Torres J Díaz-Fuentes G. Acute colonic pseudo-obstruction caused by dexmedetomidine: a case report and literature review. Am J Case Rep. (2019) 20:278–84. doi: 10.12659/AJCR.913645, PMID: 30826812 PMC6410604

[88] Zheng Y Li Z Zhou Q. Diagnosis of small intestinal microperforation by cell morphology detection in abdominal puncture fluid: a case report. Int J Surg Case Rep. (2024) 115:109316. doi: 10.1016/j.ijscr.2024.109316, PMID: 38306870 PMC10847150

[89] Chen H Kuo J Uen Y Sun D. Liver abscess secondary to fish bone migration from the duodenum. ANZ J Surg. (2011) 81:206–6. doi: 10.1111/j.1445-2197.2010.05665.x, PMID: 21342410

[90] Tepelidis C Fotiadis P Permekerlis A Karastergiou T Kouridakis P. Descending colon perforation due to ingestion of foreign body. Cureus. (2023) 15:e47479. doi: 10.7759/cureus.47479, PMID: 38022202 PMC10663047

[91] Ma T Zheng W An B Xia Y Chen G. Small bowel perforation secondary to foreign body ingestion mimicking acute appendicitis: case report. Medicine. (2019) 98:e16489. doi: 10.1097/MD.0000000000016489, PMID: 31348257 PMC6709264

[92] Coulier B Tancredi MH Ramboux A. Spiral CT and multidetector-row CT diagnosis of perforation of the small intestine caused by ingested foreign bodies. Eur Radiol. (2004) 14:1918–25. doi: 10.1007/s00330-004-2430-1, PMID: 15378256

[93] Chaugale SB Singhal V Kapoor D Singh A. Gastrointestinal complications (gangrene or perforation) after corona virus disease 2019—a series of ten patients. Indian J Gastroenterol. (2022) 41:307–12. doi: 10.1007/s12664-021-01218-z, PMID: 35471720 PMC9038998

[94] Ward MA Tews MC. Small bowel perforation secondary to fish bone ingestion managed non-operatively. J Emerg Med. (2012) 43:e295–8. doi: 10.1016/j.jemermed.2010.05.039, PMID: 20692785

[95] Lim BK Siew EP. Swallowed fish bone expelled spontaneously after perforating the small bowel. Ann Acad Med Singap. (2011) 40:475–6. doi: 10.47102/annals-acadmedsg.V40N10p475, PMID: 22206060

[96] Essrani R Hickey P Shah H. Denture misadventure: the tooth about your colon. Cureus. (2019) 11:e3890. doi: 10.7759/cureus.3890, PMID: 30911447 PMC6424550

[97] Buck E Lee S Stone LS Cerruti M. Protein adsorption on surfaces functionalized with COOH groups promotes anti-inflammatory macrophage responses. ACS Appl Mater Interfaces. (2021) 13:7021–36. doi: 10.1021/acsami.0c16509, PMID: 33539069

[98] Kong W Du Q Xin Z Cao G Liu D Wei Y. Percutaneous fully endoscopic surgical management of the ruptured epidural catheter: rescue of the novice anesthesiologist from his dilemma. Front Surg. (2022) 9:915133. doi: 10.3389/fsurg.2022.915133, PMID: 36303856 PMC9592838

[99] Poretti D Pescatori LC Mauri G Sconfienza LM Brambilla G. Inferior vena cava septic thrombosis due to gut perforation after accidental toothpick ingestion. BJR Case Rep. (2017) 3:20150522. doi: 10.1259/bjrcr.20150522, PMID: 30363322 PMC6159286

[100] Cull JN Jacobson DL Lau GA Cartwright PC Wallis MC Skarda D. Internal hernia with volvulus after major abdominal reconstructions in pediatric urology—an infrequently reported and potentially devastating complication. J Pediatr Urol. (2023) 19:402.e1–7. doi: 10.1016/j.jpurol.2023.04.030, PMID: 37179198 PMC10524189

[101] Zhang Z Yang M Zhang R. Radiographic grid for locating foreign bodies in maxillofacial emergency trauma. BMC Oral Health. (2024) 24:46. doi: 10.1186/s12903-023-03807-0, PMID: 38191426 PMC10775646

[102] Magalhães-Costa P Carvalho L Rodrigues JP Túlio MA Marques S Carmo J. Endoscopic management of foreign bodies in the upper gastrointestinal tract: an evidence-based review article. GE Port J Gastroenterol. (2016) 23:142–52. doi: 10.1016/j.jpge.2015.09.002, PMID: 28868450 PMC5580008

[103] Yoo DR Im CB Jun BG Seo HI Park JK Lee SJ. Clinical outcomes of endoscopic removal of foreign bodies from the upper gastrointestinal tract. BMC Gastroenterol. (2021) 21:385. doi: 10.1186/s12876-021-01959-3, PMID: 34666708 PMC8524826

[104] Nishino T Shinzato T Uramatsu T Obata Y Arai H Hayashida T. Bacterial peritonitis due to duodenal perforation by a fish bone in an elderly peritoneal dialysis patient. Intern Med. (2012) 51:1715–9. doi: 10.2169/internalmedicine.51.7286, PMID: 22790132

[105] Yamamoto H. Fifteen years since the advent of double-balloon endoscopy. Clin Gastroenterol Hepatol. (2017) 15:1647–50. doi: 10.1016/j.cgh.2017.08.018, PMID: 28826678

[106] Ito T Shimatani M Masuda M Nakamaru K Mitsuyama T Fukata N. Efficacy and safety of endoscopic stent placement for afferent loop obstruction using a short double-balloon endoscopy. DEN Open. (2023) 3:e154. doi: 10.1002/deo2.154, PMID: 35898829 PMC9307746

[107] Hendel JW Vilmann P Jensen T. Double-balloon endoscopy: who needs it? Scand J Gastroenterol. (2008) 43:363–7. doi: 10.1080/00365520701799468, PMID: 18266178

[108] Watanabe M Kou T Nishikawa Y Sakuma Y Kumagai N Oda Y. Perforation of the sigmoid colon by an ingested fish bone. Intern Med. (2010) 49:1041–2. doi: 10.2169/internalmedicine.49.3438, PMID: 20519824

[109] Fang C Ye L Mao X Zhang J. Endoscopic treatment of a sigmoid perforation caused by an ingested fish bone. Endoscopy. (2017) 49:E82–3. doi: 10.1055/s-0042-124500 28142158

[110] Garbati P Picco C Magrassi R Signorello P Cacopardo L Dalla Serra M. Targeting the gut: a systematic review of specific drug nanocarriers. Pharmaceutics. (2024) 16:431. doi: 10.3390/pharmaceutics16030431, PMID: 38543324 PMC10974668

[111] Dev S Pokhrel KM Mulmi U Devkota S Dev B Bhattarai A. Chicken bone-induced ileal perforation peritonitis mimicking duodenal perforation peritonitis: a case report. Ann Med Surg. (2012) 85:6202–5. doi: 10.1097/MS9.0000000000001404 PMC10718394 38098546

[112] Taguchi T Kitagawa H. Fish bone perforation. N Engl J Med. (2019) 381:762–2. doi: 10.1056/NEJMicm1900442, PMID: 31433923

[113] Nagy AG James D. Diagnostic laparoscopy. Am J Surg. (1989) 157:490–3. doi: 10.1016/0002-9610(89)90642-9, PMID: 2523669

[114] Dellino M Cerbone M Laganà AS Vitagliano A Vimercati A Marinaccio M. Upgrading treatment and molecular diagnosis in endometrial cancer-driving new tools for endometrial preservation? Int J Mol Sci. (2023) 24:9780. doi: 10.3390/ijms24119780, PMID: 37298731 PMC10253366

[115] Sermonesi G Tian BWCA Vallicelli C Abu-Zidan FM Damaskos D Kelly MD. Cesena guidelines: WSES consensus statement on laparoscopic-first approach to general surgery emergencies and abdominal trauma. World J Emerg Surg. (2023) 18:57. doi: 10.1186/s13017-023-00520-9, PMID: 38066631 PMC10704840

[116] Zhang S Wang Q Feng Y Zhang G Chen Y Zheng W. Clip or tattooing: a comparative study for preoperative colon cancer endoscopic localization. Front Oncol. (2022) 12:846900. doi: 10.3389/fonc.2022.846900, PMID: 35280761 PMC8916562

[117] Alemanno G Prosperi P Di Bella A Socci F Batacchi S Peris A. Bedside diagnostic laparoscopy for critically ill patients in the intensive care unit: retrospective study and review of literature. J Minimal Access Surg. (2019) 15:56–62. doi: 10.4103/jmas.JMAS_232_17, PMID: 29483381 PMC6293667

[118] Dural AC Çelik MF Yiğitbaş H Akarsu C Doğan M Alış H. Laparoscopic resection and intracorporeal anastomosis of perforated small bowel caused by fish bone ingestion. Turk J Trauma Emerg Surg. (2016) 22:572–4. doi: 10.5505/tjtes.2016.88137, PMID: 28074453

[119] Li M Liu Q Zhang T Gao Y Tan X Yin Z. Evaluating the learning curve of robotic radical antegrade modular pancreatosplenectomy: a retrospective cohort study. Int J Surg. (2022) 101:106612. doi: 10.1016/j.ijsu.2022.106612, PMID: 35447362

[120] Gage D Neilson T Pino MG Eiferman D Knight-Davis J. Establishment of a 24/7 robotic acute care surgery program at a large academic medical center. Surg Endosc. (2024) 38:4663–9. doi: 10.1007/s00464-024-11036-x, PMID: 38981880 PMC11289342

[121] Ceccarelli G Catena F Avella P Tian BW Rondelli F Guerra G. Emergency robotic surgery: the experience of a single center and review of the literature. World J Emerg Surg. (2024) 19:28. doi: 10.1186/s13017-024-00555-6, PMID: 39154016 PMC11330055

[122] Yu B Yeo IH Park JY. Life-threatening esophageal perforation due to blister pack ingestion. Am J Emerg Med. (2023) 65:219.e1–3. doi: 10.1016/j.ajem.2022.12.031, PMID: 36599774

[123] Irwin RS Ashba JK Braman SS Lee HY Corrao WM. Food asphyxiation in hospitalized patients. JAMA. (1977) 237:2744–5. doi: 10.1001/jama.1977.03270520054024 577229

[124] Rodríguez-Hermosa JI Ruiz-Feliú B Roig-García J Albiol-Quer M Planellas-Giné P Codina-Cazador A. Lethal intestinal perforation after foreign body ingestion in a superobese patient. Obes Surg. (2009) 19:1183–5. doi: 10.1007/s11695-008-9667-8, PMID: 18719967

[125] Cho SH Lee S Park JI La Yang Y Kim SR Ahn J. Age-associated spinal stenosis in the turquoise killifish. iScience. (2023) 26:107877. doi: 10.1016/j.isci.2023.107877, PMID: 37810235 PMC10550727

[126] Shishido T Suzuki J Ikeda R Kobayashi Y Katori Y. Characteristics of fish-bone foreign bodies in the upper aero-digestive tract: the importance of identifying the species of fish. PLoS One. (2021) 16:e0255947. doi: 10.1371/journal.pone.0255947, PMID: 34403441 PMC8370622

[127] Lu Y Shi C Jin X He J Yin Z. Domestication of farmed fish via the attenuation of stress responses mediated by the hypothalamus-pituitary-inter-renal endocrine axis. Front Endocrinol. (2022) 13:923475. doi: 10.3389/fendo.2022.923475, PMID: 35937837 PMC9353172

[128] Chen J Jayachandran M Bai W Xu B. A critical review on the health benefits of fish consumption and its bioactive constituents. Food Chem. (2022) 369:130874. doi: 10.1016/j.foodchem.2021.130874, PMID: 34455321

[129] Maulu S Musuka CG Molefe M Ngoepe TK Gabriel NN Mphande J. Contribution of fish to food and nutrition security in Southern Africa: challenges and opportunities in fish production. Front Nutr. (2024) 11:1424740. doi: 10.3389/fnut.2024.1424740, PMID: 39698239 PMC11653585

[130] Fiorella KJ Okronipa H Baker K Heilpern S. Contemporary aquaculture: implications for human nutrition. Curr Opin Biotechnol. (2021) 70:83–90. doi: 10.1016/j.copbio.2020.11.014, PMID: 33445136

[131] Naylor RL Hardy RW Buschmann AH Bush SR Cao L Klinger DH. A 20-year retrospective review of global aquaculture. Nature. (2021) 591:551–63. doi: 10.1038/s41586-021-03308-6, PMID: 33762770

[132] Bennett G Bardon LA Gibney ER. A comparison of dietary patterns and factors influencing food choice among ethnic groups living in one locality: a systematic review. Nutrients. (2022) 14:941. doi: 10.3390/nu14050941, PMID: 35267916 PMC8912306

[133] Asche F Yang B Gephart JA Smith MD Anderson JL Camp EV. China’s seafood imports-not for domestic consumption? Science. (2022) 375:386–8. doi: 10.1126/science.abl4756 35084951

[134] Xu H Wu T Budhathoki M Fang DS Zhang W Wang X. Consumption patterns and willingness to pay for sustainable aquatic food in China. Foods. (2024) 13:2435. doi: 10.3390/foods13152435, PMID: 39123626 PMC11312269

[135] Sasaki K Motoyama M Watanabe G Nakajima I. Meat consumption and consumer attitudes in Japan: an overview. Meat Sci. (2022) 192:108879. doi: 10.1016/j.meatsci.2022.108879, PMID: 35687968

[136] Nomura M Yamaguchi M Inada Y Nishi N. Current dietary intake of the Japanese population in reference to the planetary health diet-preliminary assessment. Front Nutr. (2023) 10:1116105. doi: 10.3389/fnut.2023.1116105, PMID: 37077901 PMC10106588

[137] Yoshikawa E Nishi D Matsuoka YJ. Association between frequency of fried food consumption and resilience to depression in Japanese company workers: a cross-sectional study. Lipids Health Dis. (2016) 15:156. doi: 10.1186/s12944-016-0331-3, PMID: 27633655 PMC5025553

[138] Aljassim N Ostini R. Health literacy in rural and urban populations: a systematic review. Patient Educ Couns. (2020) 103:2142–54. doi: 10.1016/j.pec.2020.06.007, PMID: 32601042

[139] Wu CW Chiu YW. Unintentional fish bone ingestion causing perforation of small intestine. Intern Emerg Med. (2021) 16:1371–2. doi: 10.1007/s11739-020-02519-5, PMID: 33025532

[140] Yasuda T Kawamura S Shimada E Okumura S. Fish bone penetration of the duodenum extending into the pancreas: report of a case. Surg Today. (2010) 40:676–8. doi: 10.1007/s00595-009-4110-x, PMID: 20582523

[141] Yuan F Liu Y Wen M. Fish bone perforation of the transverse colon. Intern Emerg Med. (2024) 19:1771–2. doi: 10.1007/s11739-024-03591-x, PMID: 38607541

[142] Huang YH Siao FY Yen HH. Pre-operative diagnosis of pancreatic abscess from a penetrating fish bone. QJM. (2013) 106:955–6. doi: 10.1093/qjmed/hcs166, PMID: 22927535

[143] Jallali M Zenati H Korbi A Chaouch MA Jabra SB Korbi I. Small bowel perforation with ingestion of a fish bone: case report. Pan Afr Med J. (2024) 48:94. doi: 10.11604/pamj.2024.48.94.42796, PMID: 39492854 PMC11530384

[144] Dugas B Bernard B Odet E Naouri A Bernard P Valette P. Bad dentition and risk of jejunal perforation by fish bone. J Am Geriatr Soc. (2005) 53:1632–3. doi: 10.1111/j.1532-5415.2005.53487_2.x, PMID: 16137303

[145] Guillén-Paredes MP Lirón-Ruiz R Torralba-Martínez JA Martín-Lorenzo JG Aguayo-Albasini JL. Intestinal perforation caused by incidental ingestion of a fish bone: value of CT in the diagnosis. Rev Esp Enferm Dig. (2010) 102:569–70. doi: 10.4321/S1130-01082010000900016, PMID: 20883081

[146] Chandrasinghe PC Pathirana CK. Laparoscopically detected and nonsurgically managed ileal perforation by an ingested fish bone: a case report. J Med Case Rep. (2015) 9:43. doi: 10.1186/s13256-015-0526-7, PMID: 25888949 PMC4349716

[147] Wu CX. Rare case of omentum-wrapped abscess caused by a fish bone penetrating the terminal ileum. World J Gastroenterol. (2014) 20:11456. doi: 10.3748/wjg.v20.i32.11456 25170236 PMC4145790

[148] Zhao SG Xu JJ Xu L Zheng JF Zhou ZC Jiang LQ. Ileal perforation caused by a fish bone shortly after drug-eluting stent implantation for acute myocardial infarction. J Int Med Res. (2019) 47:2709–15. doi: 10.1177/0300060519842778, PMID: 31014143 PMC6567709

[149] Mutlu A Uysal E Ulusoy L Duran C Selamoglu D. A fish bone causing ileal perforation in the terminal ileum. Turk J Trauma Emerg Surg. (2012) 18:89–91. doi: 10.5505/tjtes.2012.90912, PMID: 22290059

[150] Hassani KIM Toughrai I. Péritonite par perforation grêlique secondaire à une arête de poisson. Pan Afr Med J. (2013) 15:107. doi: 10.11604/pamj.2013.15.107.3025 24244793 PMC3828070

[151] Fantola G Cenedese A Barth X Monneuse O. Laparoscopic diagnosis and treatment of small bowel perforation by fish bone. ANZ J Surg. (2011) 81:205–6. doi: 10.1111/j.1445-2197.2010.05664.x, PMID: 21342408

[152] Daniele L Elliott D Wong MS Free J. Perforation of Meckel’s diverticulum by an intact fish bone: a case report and literature review. ANZ J Surg. (2017) 87:E206–7. doi: 10.1111/ans.13125, PMID: 25924861

[153] Zhou W Ding L Dong T Liu X. Unusual case of acute appendicitis with perforation caused by an ingested fish bone. Asian J Surg. (2024) 47:1421–2. doi: 10.1016/j.asjsur.2023.11.111, PMID: 38030492

[154] Ishimura T Takenaka A Sakai Y Fujii T Jo Y Fujisawa M. Hydronephrosis caused by intra-abdominal abscess from cecal perforation by an ingested fish bone. Int J Urol. (2006) 13:1350–1. doi: 10.1111/j.1442-2042.2006.01552.x, PMID: 17010018

[155] Yamamoto M Yamamoto K Sasaki T Fukumori D Yamamoto F Igimi H. Successfully treated intra-abdominal abscess caused by fish bone with perforation of ascending colon: a case report. Int Surg. (2015) 100:428–30. doi: 10.9738/INTSURG-D-14-00163.1, PMID: 25785322 PMC4370530

[156] Chiu JJ Chen TL Zhan YL. Perforation of the transverse colon by a fish bone: a case report. J Emerg Med. (2009) 36:345–7. doi: 10.1016/j.jemermed.2007.11.007, PMID: 18281182

[157] Saleem A Alenezi S Abdulbaqi S Saud A Al-Shadidi N. Multiple abdominopelvic abscesses caused by fishbone: a case report of rare etiology and literature review. Int J Surg Case Rep. (2023) 110:108608. doi: 10.1016/j.ijscr.2023.108608, PMID: 37579633 PMC10448268

[158] Cho MK. Fish bone migration to the urinary bladder after rectosigmoid colon perforation. World J Gastroenterol. (2014) 20:7075. doi: 10.3748/wjg.v20.i22.7075, PMID: 24944504 PMC4051954

[159] Endo S Minakuchi H Yoshida T. Peritoneal dialysis patient having fish bone-induced colon perforation. Clin Exp Nephrol. (2019) 23:717–8. doi: 10.1007/s10157-018-1670-3, PMID: 30421261

[160] Hawkyard A Armitage G. Peritonitis due to perforation of the bowel by foreign bodies. BMJ. (1931) 1:577–8. doi: 10.1136/bmj.1.3665.577, PMID: 20776100 PMC2314015

[161] Yamashita K Komohara Y Uchihara T Arima K Uemura S Hanada N. A rare case of perforation of a colorectal tumor by a fish bone. Clin J Gastroenterol. (2022) 15:598–602. doi: 10.1007/s12328-022-01622-8, PMID: 35312955

